# Synchronizing Tunable Luminescence and Shape Morphing in a Metal Nanocluster–Enabled Hydrogel Platform

**DOI:** 10.1002/adma.73386

**Published:** 2026-05-14

**Authors:** Hongbin Lin, Zening Huang, Yongshi He, Hongda Wei, Zhucheng Yang, Zhiqiang Hu, Yang Zhou, Xiaorong Song, Sanyang Han, Jianping Xie

**Affiliations:** ^1^ Institute of Biopharmaceutical and Health Engineering Tsinghua Shenzhen International Graduate School (SIGS) Tsinghua University Shenzhen China; ^2^ Department of Chemical and Biomolecular Engineering National University of Singapore Singapore Singapore; ^3^ Department of Gastric Surgery Fujian Medical University Union Hospital Fuzhou China; ^4^ MOE Key Laboratory For Analytical Science of Food Safety and Biology and State Key Laboratory of Photocatalysis on Energy and Environment College of Chemistry Fuzhou University Fuzhou China

**Keywords:** aggregation induced emission, metal nanoclusters, nanocluster‐gel platform, organic solvent leakage, solvent detection

## Abstract

Soft hydrogels capable of simultaneous shape morphing and optical modulation under a single stimulus hold promise for next‐generation intelligent materials. However, most systems rely on complex architectures or multi‐component triggers, limiting integration and responsiveness. Here, we develop a structurally homogeneous nanocluster–gel platform by embedding water‐soluble gold nanoclusters (AuNCs) into a polyacrylamide matrix, enabling synchronized mechanical deformation and tunable luminescence in response to solvent polarity. Upon exposure to organic solvents, the gel rapidly contracts (<5 s), while spatial confinement of AuNCs within the polymer boosts quantum yield from 6.05% to 22.83%. The emission profile is programmable by varying cluster size, highlighting AuNCs as structurally tunable emission modulators. Kinetic analysis reveals non‐Fickian swelling/deswelling—governed by solvent diffusion and polymer relaxation—that synergistically modulates emission. This enables real‐time, quantitative optical detection of solvent gradients (R^2^ = 0.996) with >97% reversibility over 10 cycles. The integrated responsiveness is demonstrated in a biomimetic lotus‐shaped gel that folds and dims upon water uptake, and is further validated in a leakage scenario where the hydrogel serves as a pipe encapsulation film, achieving rapid sealing and visual monitoring of solvent leakage. This work establishes a nanocluster–gel platform that unifies photophysics and actuation for adaptive, self‐indicating soft systems.

## Graphical abstract

A nanocluster–gel platform is developed to synchronously integrate tunable luminescence and shape morphing under a single stimulus, with emission programmability encoded through cluster size modulation (Au_10_ → Au_18_). Solvent‐induced shape–optical transitions are visualized in a lotus‐shaped gel that dynamically folds and dims, enabling rapid (< 5 s) sealing and polarity‐triggered luminescence for solvent leakage suppression and detection.

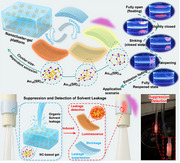

## 1 Introduction

Stimuli‐responsive hydrogels are a quintessential class of soft smart materials capable of reversible shape or volume changes upon environmental triggers such as temperature [1, 2], pH [3], light [4], or solvent composition [5]. They hold promise for applications ranging from soft robotics and actuators to sensors and adaptive optics [6, 7], wherein a single material can undergo programmed deformation. A central challenge, however, is integrating mechanical deformation with an optical output under one stimulus [8, 9]. In conventional designs, the shape‐morphing function of hydrogels has rarely been coupled intrinsically with a simultaneous color or photoluminescence signaling, a capability that is vital for visual sensors, camouflage, and information encryption [10, 11]. Achieving synchronized deformation and optical readout in response to one gentle stimulus remains difficult due to materials and design constraints.

Recent strategies have begun to bridge this gap by incorporating functional chromophores into hydrogel networks. Aggregation‐induced emission (AIE) fluorophores [12], for example, have been incorporated into hydrogels to induce fluorescence through solvent‐triggered molecular packing or polymer collapse. Photochromic molecules like spiropyrans have been covalently embedded to impart light‐triggered color shifts alongside light‐induced mechanical bending [13, 14]. Supramolecular approaches have also been explored [15, 16], for instance, using host–guest interactions or mechanophore linkages that produce a color change when the network is strained or reconfigured. Each of these approaches demonstrates the feasibility of coupling optical and mechanical responses, but they often come with drawbacks. Many require multi‐component architectures such as separate dye‐doped layers or hybrid networks, which complicate material fabrication and can lead to slower, sequential responses (one component responding before triggering the other) [3, 17, 18]. Others rely on intense or specific stimuli (e.g., UV light or high heat) or complex chemistries that limit their practicality [19, 20]. Overall, existing solutions tend to sacrifice structural simplicity or response speed, highlighting the need for a more integrated, efficient strategy.

Metal nanoclusters (NCs) [21, 22, 23] have emerged as a unique class of luminescent building blocks that can address these challenges. Unlike bulk nanoparticles, metal NCs (comprising only a few to tens of atoms, e.g., Au_18_ and Au_15_) exhibit molecule‐like photoluminescence that is highly tunable by their metal core and surface ligands [24, 25, 26, 27]. They are inherently sensitive to their environment; subtle changes in solvent polarity [28, 29], cluster packing [30], or ligand interactions [31, 32] can significantly shift their emission intensity and wavelength. For instance, a recent impressive breakthrough has revealed that core symmetry breaking modulates electron–acoustic phonon coupling under environmental pressure [33], thereby tuning emission. Notably, many AuNCs display aggregation‐induced or confinement‐enhanced emission [34], in stark contrast to conventional organic dyes that typically quench upon aggregation [35, 36]. When dispersed in solution, these NCs often fluoresce weakly (due to nonradiative relaxations) [28, 34], but upon being immobilized in a rigid matrix, their luminescence is dramatically boosted as vibrational deactivation pathways are suppressed [37, 38]. This property makes metal NCs ideal for embedding in hydrophilic polymer networks [39, 40, 41]: the hydrogel's swollen or shrunken state can directly modulate the NC's emissive behavior. Moreover, AuNCs are generally robust, water‐soluble (with appropriate ligands) and biocompatible, offering advantages in stability and modular tunability over traditional organic fluorophores or AIEgens that may require elaborate synthesis for each new color [42, 43, 44]. These features suggest that NC‐doped hydrogels could provide a single‐component (yet composite) system wherein the polymer matrix provides mechanical actuation and the NCs provide a responsive optical signal, all driven by the same stimulus.

**FIGURE 1:**
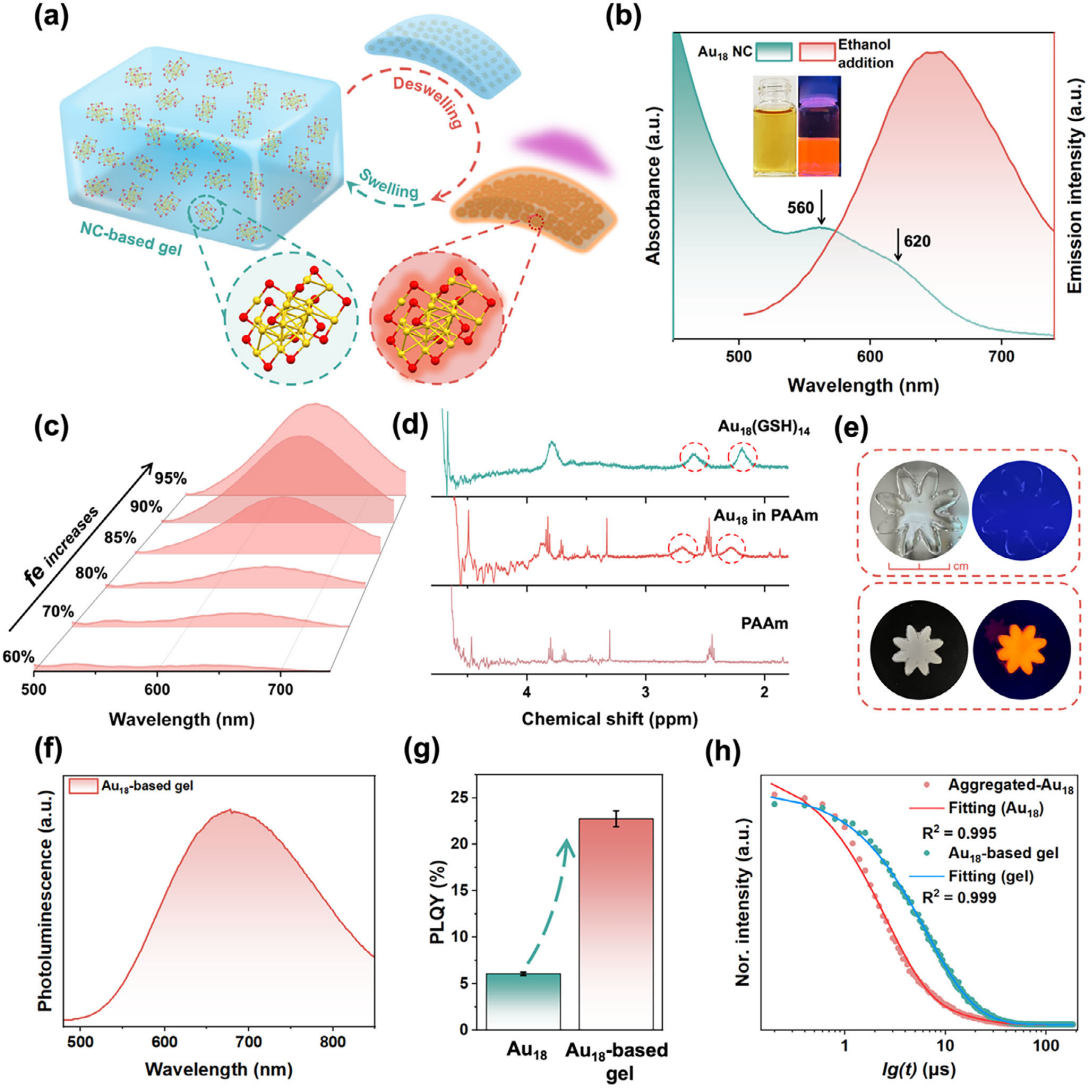
(a) Schematic illustration of the behavior of gel embedded with gold nanoclusters (NC) under organic solvents: shrinkage and luminescence. (b) UV–vis spectrum of synthesized Au_18_(GSH)_14_ (GSH = glutathione) NCs (hereafter abbreviated as Au_18_), and emission spectrum of aggregated Au_18_ in an ethanol/water mixture (volume fraction of ethanol, *fe* = Vol_ethanol_/vol_water+ethanol_ at 95%), left and right instes (digital photos of synthesized Au_18_ solution and fluorescent image of Au_18_ solution after ethanol treatment). (c) Emission spectra of Au_18_ under different *fe* values. (d) Partial enlarged ^1^H NMR spectra of Au_18_(GSH)_14_ in D_2_O (∼0.23 mm), Au_18_ in polyacrylamide (PAAm) monomer solution, and Au_18_‐free PAAm monomer solution. (e) Digital photos and fluorescent images of swollen Au_18_‐based gel in the top panel, and deswollen Au_18_‐based gel in the bottom panel. (f) Emission spectra of deswollen Au_18_‐based gel after soaking in anhydrous ethanol solution. (g) The photoluminescent quantum yield comparison between aggregated Au_18_ NCs and deswollen Au_18_‐based gel. (h) Photoluminescent lifetime and fitting results of aggregated Au_18_ and deswollen Au_18_‐based gel. All emission spectra were recorded with an excitation wavelength of 365 nm.

In this regard, we developed a structurally homogeneous nanocluster–gel platform by incorporating water‐soluble AuNCs into polyacrylamide (PAAm) topological gel networks (Figure 1a). Solvent polarity acts as a single, mild trigger that synchronously modulates gel deformation and luminescence via dual‐scale mechanisms: at the molecular level, solvent‐induced desolvation of NC ligands and polymer densification collectively activate aggregation‐enhanced luminescence; concurrently, polarity‐governed polymer–solvent interactions drive volumetric transitions of the gel network (Flory–Huggins parameter χ > 0.5) [45, 46]. These coupled processes establish a unified actuation–emission response pathway from nanoscopic reorganization to macroscopic deformation, thereby allowing environmental stimuli to simultaneously trigger both mechanical deformation and optical emission in a coupled, reversible manner. Within this unified framework, the emission characteristics can be readily tailored via cluster size, highlighting the modularity of the platform. Such capabilities are vividly demonstrated in a biomimetic lotus‐shaped construct that encodes hydration states via coordinated shape transformation and emission modulation. Further validated in a practical solvent leakage scenario with autonomous sealing and visual optical feedback, this system introduces a minimalistic yet multifunctional nanocluster–gel strategy for adaptive sensing, soft logic, and intelligent fluidic interfaces.

## 2 Results and Discussion

In our study, we first synthesized water‐soluble Au_18_(GSH)_14_ (GSH = glutathione) NCs (hereafter abbreviated as Au_18_) according to a previous protocol [47]. UV–vis absorption spectra exhibit absorption peaks at 560 and 620 nm (Figure 1b), which is in agreement with those of Au_18_. The resulting product was characterized by a light brown solution, indicating the successful synthesis of Au_18_ (left inset of Figure 1b) [47]. By introducing ethanol into the solution, a strong, deep orange emission centered at 653 nm was observed (Figure 1b). Upon further deliberate adjustment of volume fraction in the ethanol and water mixture (*fe* = Vol_ethanol_/vol_water+ethanol_), the emission intensity of Au_18_ species is significantly enhanced as the *fe* value increases from 60% to 95% (Figure 1c). At *fe* of 95%, the photoluminescent quantum yield (PLQY) of the product was measured to be 6.05% (Figure 1g), and hence these results present very typical AIE characteristics of metal NCs. Subsequently, we embedded the as‐prepared Au_18_ in the PAAm gel. In a typical experiment, the synthesized NC solution was initially added to a mixture of acrylamide and *N,N'*‐methylenebisacrylamide at a mass ratio of 19:1 to form the NC‐based monomer solution. Then, the gel initiator, ammonium persulfate, and *N,N,N′,N′*‐tetramethylethylenediamine were added sequentially to the monomer solution under vigorous stirring. After approximately 30 min, gelation was observed, suggesting the successful formation of an Au_18_‐based gel (Figure 1a). As shown in Figure 1d, compared to those in the free Au_18_ solution, the ^1^H NMR signals of Au_18_ in the PAAm monomer mixture exhibit obvious peak broadening due to restricted ligand motion in the monomer environment, suggesting partial immobilization of Au_18_ within the forming polymer network [37, 48]. The resulting Au_18_‐based gels are characterized by a transparent and flexible state (Figure 1e).

We next soaked the prepared Au_18_‐based gel in an anhydrous ethanol solution for approximately two hours. The gels became substantial shrinkage, accompanied by a dramatic increase in photoluminescence (Figure 1e,f). PLQY of the resulting gels was improved to 22.83% (Figure 1g). This observation suggests that the mesoporous confinement effects of gel matrix effectively stabilize quantum‐size distributions of AuNCs, suppressing the performance degradation issue in the liquid states. Within the gel matrix, the tight and ordered structure promotes aggregation of Au_18_, enhancing inter‐cluster electronic coupling and modifying the energy band structure [49, 50]. These effects collectively lead to increased luminescence intensity and prolonged fluorescence lifetime (Figure 1e,h) due to the suppressed non‐radiative decay and enhanced radiative transitions (Table S1) [51, 52].

**FIGURE 2:**
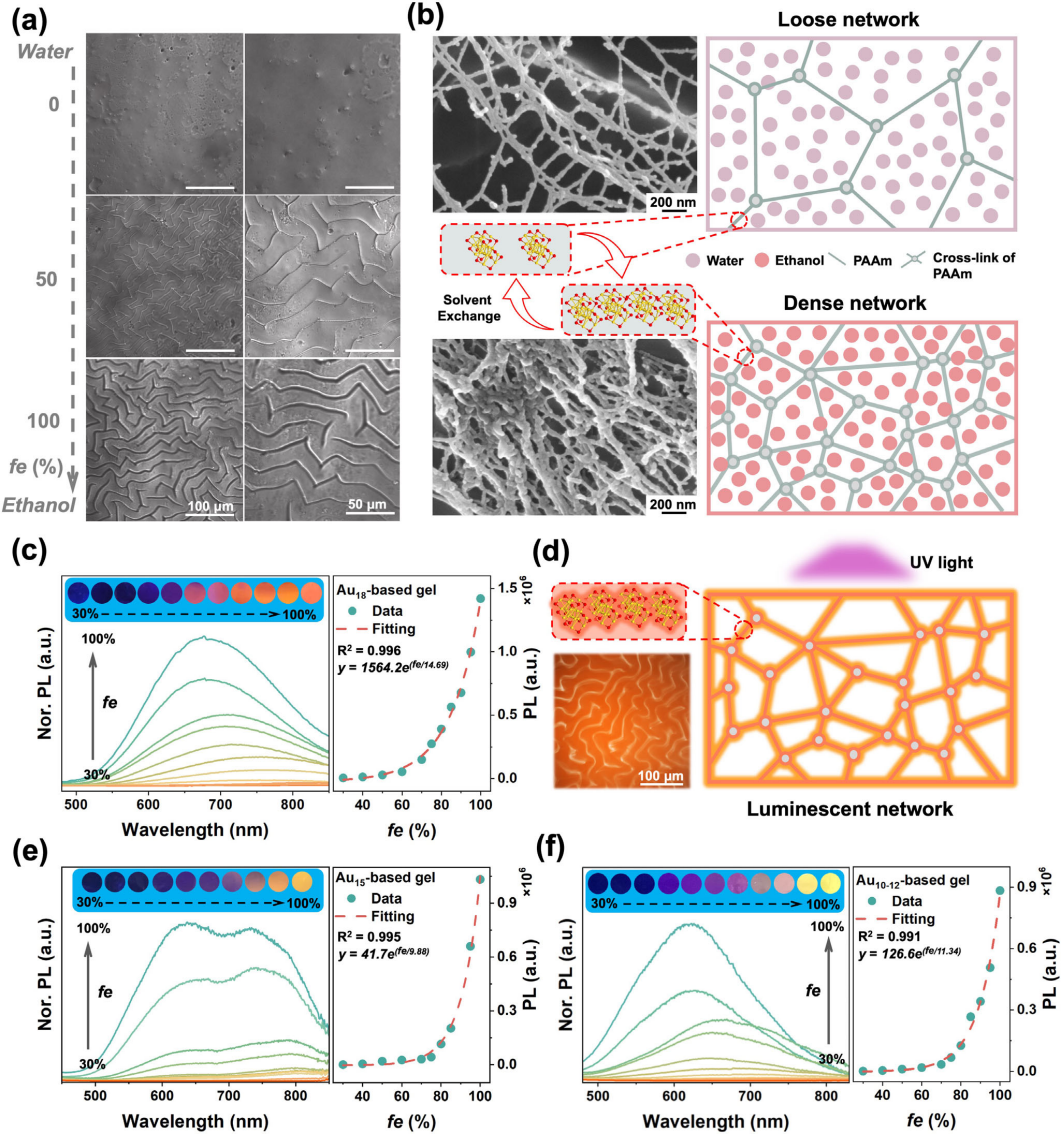
(a) Confocal images under bright field corresponding to Au_18_‐based gel treated by pure water (*fe* = 0%), at *fe* = 50%, and at anhydrous ethanol (*fe* = 100%) solution. (b) SEM images of Au_18_‐based gel under swollen state and deswollen state, as well as corresponding schematic diagrams. (c) Emission spectra of Au_18_‐based gel immersed in different concentrations of ethanol solutions as *fe* value spans from 30% to 100%, with fluorescence images under UV light displayed at the top, and the exponential growth fit of fluorescence intensity versus *fe* value. (d) Schematic illustration about the luminescent state of the dense gel network, and corresponding inverted fluorescence image. (e,f) Emission spectra of Au_15_‐ and Au_10‐12_‐based gels across the same *f_e_* range (30%–100%), each with fluorescence images under UV and exponential fits of emission intensity versus *f_e_*. Excitation wavelength for all emission spectra: 365 nm.

Structure conformation change of Au_18_‐based gel is highly dependent on the ethanol‐to‐water volume ratio. We next observe the surface morphology by deliberately modulating the *fe* value. Initially, in pure water (*fe* = 0), the as‐prepared gel is in a swollen state with a smooth surface morphology. When the *fe* value increases to 50%, the surface begins to wrinkle. With further increasing the *fe* value, an obvious wrinkle can be observed with *fe* value of 100% (Figure 2a), suggesting that the gel was converted to a deswollen state. To unravel the underlying mechanism, we performed scanning electron microscopic measurements to investigate the surface morphology of the swollen and deswollen gels. As shown in Figure 2b (top panel), the PAAm network in the swollen state exhibits a loose structure with relatively thin fibers. In contrast, the PAAm network becomes more compact with thicker fibers in the deswollen state (Figure 2b, bottom panel). As a result, the deswelling and shrinkage of the gel lead to a remarkable enhancement in luminescence of Au_18_ embedded in the gel (Figure 2d). Interestingly, the emission intensity exhibits a more than 260‐fold enhancement (Figure 2c, with PLQY increasing from approximately 0% to 22.83%) accompanied by progressive volume shrinkage (Figure S2) as the *fe* value increases from 30% to 100%. Notably, the emission intensity of the gel shows an excellent exponential growth relationship with the *fe* value (R^2^ = 0.996).

We attributed the enhanced emission to the synergistic effects of solvent‐responsive dynamic confinement and AIE, as clearly demonstrated in Figure 2b. When the gel is immersed in pure water, the hydrophilic PAAm network undergoes significant swelling due to strong solvent interaction, and its loose three‐dimensional topology leads to an increased distance between Au_18_ NCs (top panel of Figure 2b), which weakens the electronic coupling between clusters. Additionally, the high dielectric solvation layer (with dielectric constant ∼ 80) between water molecules with clusters induces intra‐molecular vibrational relaxation and solvent‐assisted non‐radiative energy dissipation [53], thus resulting in weak emission. Upon transferring the gel to ethanol, the sharp decrease in solvent polarity (dielectric constant ∼25.3) [54] triggers rapid deswelling of the gel network. This polarity change weakens the solvation of the hydrophilic groups on the PAAm fibers. Subsequently, the PAAm fibers rearrange through hydrophobic interactions and form a compact or dense structure (bottom panel of Figure 2b), thus shortening the inter‐cluster distance. In addition, the rigid network significantly suppresses the rotation and vibration of the ligands [37, 55, 56], resulting in a bright luminescence (Figure 2d).

Building upon the polarity‐responsive luminescence of Au_18_‐based gels, we further explored whether such emission behavior could be systematically tuned by engineering the intrinsic properties of the embedded AuNCs. Given the strong correlation between cluster size and electronic structure, we synthesized Au_15_ and Au_10‐12_ NCs (Figure S3) and incorporated them into the same PAAm gel matrix. When immersed in ethanol, the resulting gels exhibited distinctly different emission colors (Figure 2e,f), ranging from bright yellow to orange, depending on the cluster size. This size‐dependent photoluminescence behavior highlights the structural programmability of AuNCs as modular emissive units within the gel network. Moreover, it underscores the versatility of the nanocluster–gel platform, which enables customized optical responses through rational selection of cluster composition and electronic structure. Such design flexibility further suggests that the observed polarity‐responsive photoluminescent behaviours are not limited to GSH‐protected AuNCs, but can be extended to other ligand‐protected systems (e.g., 3‐mercaptopropionic acid (MPA) and 6‐mercaptohexanoic acid (MHA)‐capped AuNCs) [47, 57] as well as AgNCs [58], demonstrating the generality of this nanocluster–gel platform (Figure S4).

**FIGURE 3:**
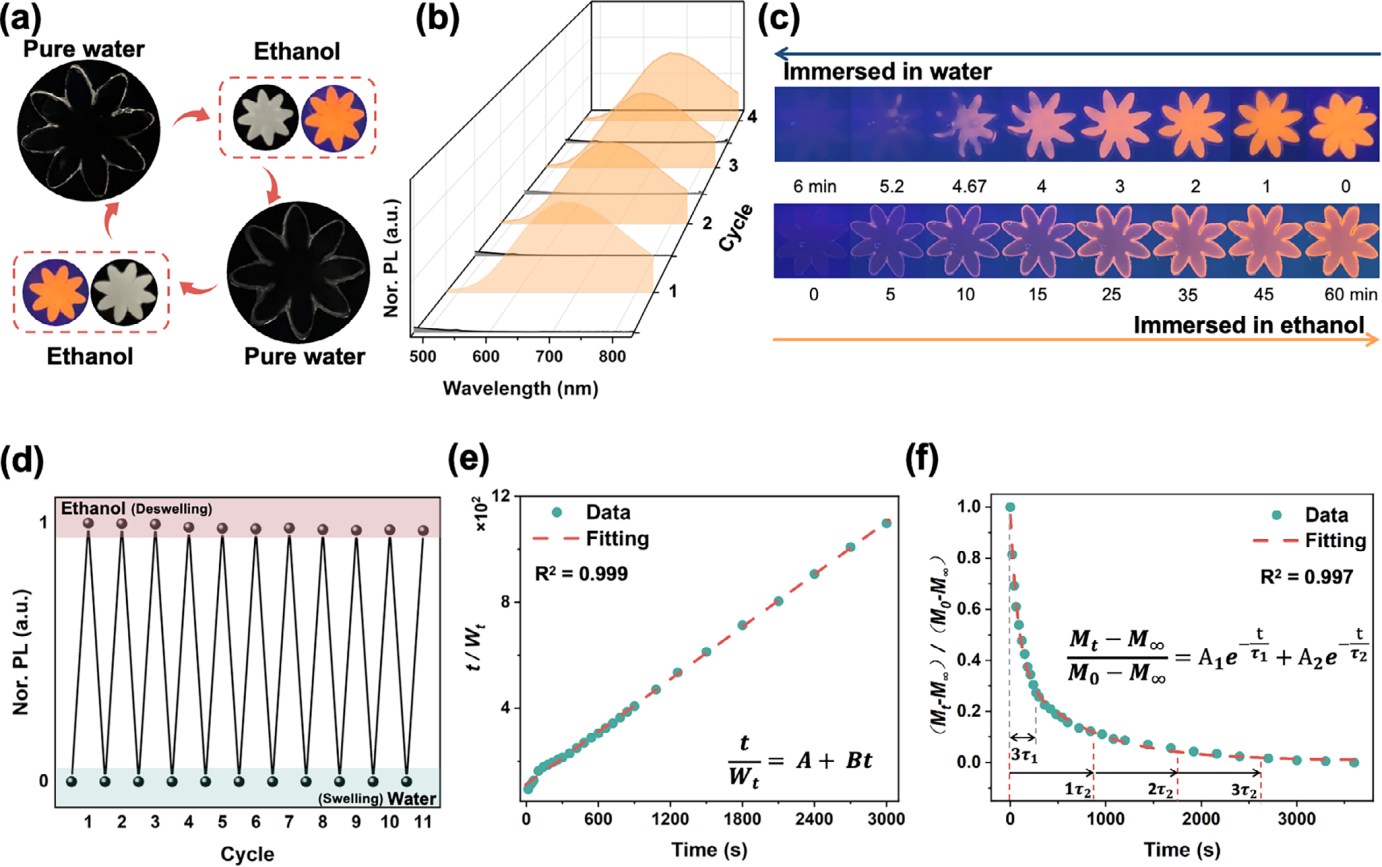
(a) Photographs and corresponding fluorescence images of the NC‐based gel under repeated solvent exchange cycles. (b) Emission spectra of the NC‐based gel over multiple swelling/deswelling cycles (λ_ex_ = 365 nm). (c) Time‐resolved fluorescence images of gel during swelling and deswelling processes. (d) Comparison of normalized maximum emission intensity across multiple swelling/deswelling cycles. (e) Second‐order kinetic fitting of gel during the swelling process. (f) Biexponential decay fitting of gel during the deswelling process.

Interestingly, beyond size‐dependent color modulation, the polarity‐triggered gel transformation also led to a remarkable enhancement in both mechanical performance and photoluminescence. As the gel transitions from a swollen state to a deswollen state (Figure 3a), tensile strength and emission of the deswollen gels were measured to be improved by over 170 and 260 times, respectively, compared to the swollen counterpart (Figure S5). Furthermore, the deswollen gel can be easily reverted to the swollen state by introducing water. After over 10 cycles, no obvious change in mechanical strength and emission intensity of the gels was observed (Figure 3b,d), demonstrating the excellent reusability of the Au_18_‐based gels. This reversible switching behavior is further supported by the time‐dependent luminescence evolution in different solvents. As shown in Figure 3c, the gel emission was gradually enhanced with increasing soaking time in ethanol, while it gradually diminished upon re‐immersion in water.

To further investigate the swelling and deswelling behavior of the as‐prepared gels [59, 60], the gel's mass variation was first monitored to characterize the amount of water retained in the gel (swelling ratio *W_t_*) to investigate this swelling process. The experimental mass data (obtained from four repeated measurements and averaged) were fitted using both first‐order and second‐order swelling kinetics [61, 62]. The fitting analysis results reveal that the first‐order model fitting shows a low correlation coefficient of R^2^ = 0.723 (Figure S6a). This discrepancy suggests that the swelling is not primarily governed by diffusion‐dominated (Fickian) behavior [63]. By contrast, the second‐order kinetic analysis offers an excellent fit (R^2^ = 0.999; Figure 3e) with the inverse of the hydrogel's initial swelling rate (*A* = 114.33) and the reciprocal of the equilibrium swelling ratio (*B* = 0.34). The theoretical equilibrium swelling ratio ($W_{\infty }^{\prime }$), calculated as the inverse of the slope (*B*), yields a value of 2.94, which is in close agreement with the experimental value of 2.76.

Unlike first‐order kinetics, where the swelling rate is directly proportional to the remaining swelling capacity, second‐order kinetics assumes the rate is proportional to the square of the unswollen capacity. This suggests that, as swelling progresses, the decline in swelling rate is more pronounced than in first‐order behavior. Such nonlinear kinetics indicate a cooperative mechanism, where both solvent diffusion into the PAAm network and polymer fiber relaxation contribute jointly to govern the swelling dynamics [64, 65]. To further confirm it, the early‐stage swelling data (*W_t_*/*W*_∞_ ≤ 60%) was fitted using Ritger‐Peppas model [66, 67, 68]. The fitting results illustrate a linear relationship between ln (*W_t_*/*W*_∞_) and *lnt*, with an *n* value of 0.7 and a high correlation coefficient of R^2^ = 0.997 (Figure S6b). This *n* value falls within the range of 0.5 and 1, indicating that the swelling process follows a non‐Fickian transport mechanism governed by the coupled effects of solvent diffusion and polymer fiber relaxation, in agreement with the second‐order swelling kinetics analyses.

Subsequently, we studied the deswelling behavior of Au_18_‐based gels by a biexponential decay model [69, 70]. As shown in Figure 3f, a high correlation coefficient (R^2^ = 0.997) results, revealing two distinct phases in the deswelling process, suggesting the co‐existence of a rapid surface diffusion phase and a slow network restructuring phase (Table S2). In the rapid phase (time constant τ_1_ = 91.02 s), the normalized swelling degree decreases from 1 to 0.274 after 3τ_1_ (273 s). This stage involves rapid water expulsion from the gel surface, leading to significant mass loss and fast contraction of the outer layer. When correlating with the gel's luminescence behavior, the initial swollen gel offers negligible luminescence due to the high degree of freedom of the PAAm fibers and the relatively loose network structure. As water molecules were expelled from the gel surface in the first 5 min (∼3τ_1_, bottom panel in Figure 3c), the PAAm fibers become increasingly confined to initiate the emission recovery process. In the slow phase (a time constant τ_2_ =  887.31s), the normalized swelling degree decreased to 0.11 within the time span of 3τ_2_ (∼45 min). This phase involves the removal of water from deeper layers and gradual PAAm fibers rearrangement. The more compact configuration improves the confinement of NCs, offering bright emission from the NCs by 45 min (∼3τ_2_) as illustrated in Figure 3c. After 60 min, the mass of the gel keeps constant and the network reached a stable state.

**FIGURE 4:**
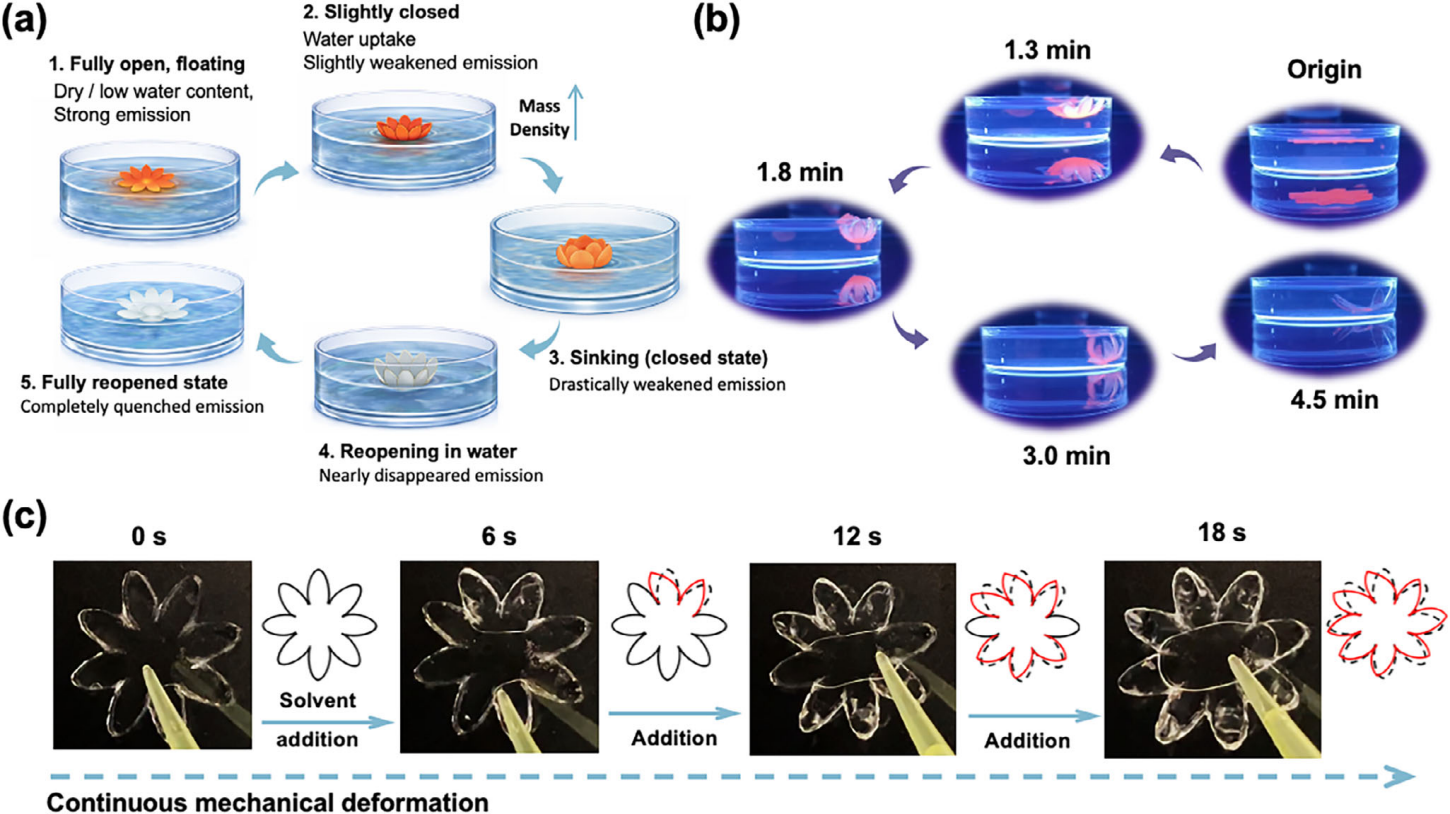
(a) Schematic showing the gel floating and emitting upon dehydration, folding and submerging during water uptake, and reopening with quenched luminescence after full rehydration. (b) Corresponding time‐lapse images capturing the dynamic shape and emission changes during the transition process under. (c) Sequential images showing time‐dependent deformation of the flower‐shaped NC‐based gel after the application of organic solvent droplets.

Motivated by the gel's reversible deswelling–emission kinetics, we next employed the pre‐formed biomimetic lotus‐shaped AuNC‐based gel to intuitively visualize its dynamic reversible responses to solvent environments. Upon dehydration, the flower structure floated on water while exhibiting bright luminescence. As the gel gradually absorbed water, the petals folded inward, accompanied by a continuous decrease in emission intensity. Eventually, the entire structure sank below the surface, fully reopened, and its luminescence disappeared (Figure 4a,b and Video S1). This transformation illustrates the developed nanocluster–gel platform's ability to convert solvent‐mediated molecular interactions into coordinated macroscopic actuation and optical modulation. Such synchronized, multi‐stage transitions underscore its potential as a modular soft‐matter framework for intuitive environmental interfaces and solvent‐adaptive logic systems.

To shed more light on our nanocluster‐gel system, we tested the generality of the as‐prepared gel in responsive to various organic solvents. In our experiment, NC‐based gels were immersed in methanol and acetone. As anticipated, a pronounced shrinkage and strong luminescence were clearly observed from the deswollen gels (Figure S7). To preliminarily evaluate the practical responsiveness of NC‐based gels toward organic solvents, a small amount of solvent was directly dropped onto the surface of a flower‐shaped gel. Surprisingly, even within 6 s, two petals began to curl inward. And the entire flower structure had nearly closed after 18 s, showing a rapid deformation response behavior (Figure 4c and Video S2). In contrast, no noticeable mechanical change occurred when an equivalent amount of pure water was dropped onto the gels (Figure S8 and Video S2). This fast, directional deformation underscores the gel's high spatiotemporal sensitivity and selectivity toward polar organic solvents, establishing a foundation for responsive material design under realistic stimuli.

**FIGURE 5:**
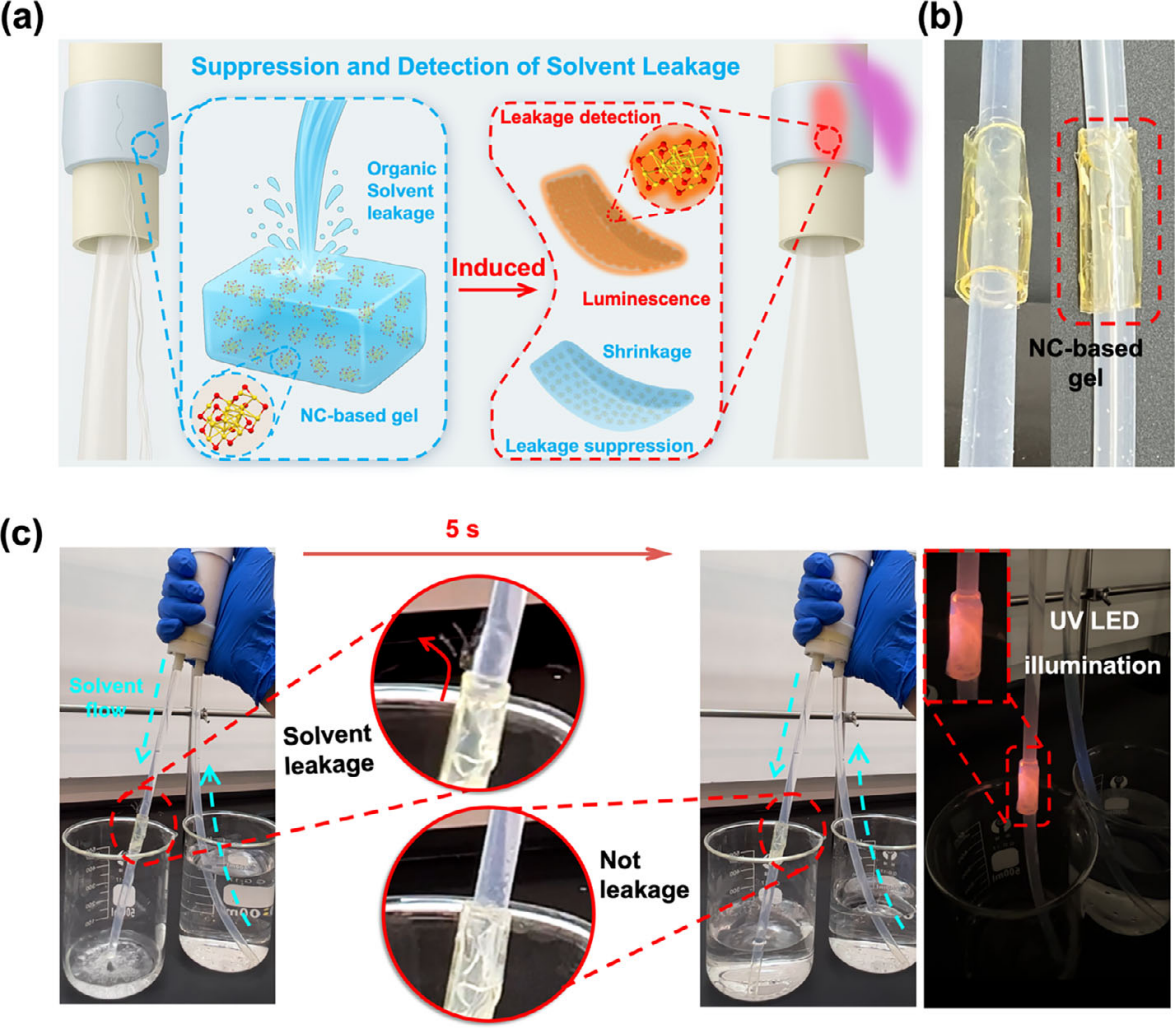
(a) Schematic illustration of the working mechanism of the dynamically responsive material system for suppressing and detecting organic solvent leakage. (b) Photographs of the NC‐based gel loosely placed over the cracked region of the pipeline. (c) Experimental photographs corresponding to each stage of gel response: solvent leakage prior to activation, rapid sealing via gel contraction, and bright luminescence indicating the leakage site under UV irradiation.

Building on these photomechanical properties, we finally explored a practical application scenario: autonomous detection and suppression of organic solvent leakage. As a proof of concept, we fabricated a pipe encapsulation thin‐film using the as‐prepared Au_18_‐based PAAm gels to repair the cracked liquid pipe. As demonstrated in Figure 5a, upon solvent leakage at the crack site, a quick shrinkage of Au_18_‐based gels was initiated to suppress the leakage, while strong luminescence could be visualized around the leakage site. In our study, a model leakage system was first constructed to simulate practical application scenarios. Specifically, a miniature peristaltic pump was used to drive organic solvent through a cracked pipeline. The Au_18_‐based gel was placed over the damaged area in a relaxed non‐tight configuration, as presented in Figure 5b. As organic solvents flow through the damaged site, we observed a quick shape‐adaptive response within just 5 s, conforming tightly to the damaged site and effectively sealing the crack, thereby suppressing further organic solvent leakage (Figure 5c, and Video S3). By comparison, in the absence of the Au_18_‐based gel, substantial leakage was observed at the damage site during solvent flow (Figure S9). At the same time, the gel at the leakage area emitted a bright luminescence under 365 nm UV LED illumination (Figure 5c and Video S3). This synchronized dual‐response capability—rapid mechanical sealing and luminescent signaling—demonstrates the viability of the developed nanocluster–gel platform as an integrated strategy for safety assurance in fluidic systems and self‐protective interfaces, highlighting its effectiveness in realistic operational conditions.

## 3 Conclusion

In summary, we present a structurally integrated nanocluster–gel platform that bridges photonic and mechanical functionalities within a single soft material system. By embedding water‐soluble AuNCs into a homogeneous polyacrylamide hydrogel network, we achieve synchronized shape morphing and programmable luminescence in response to solvent polarity. The system preserves the AIE characteristics of AuNCs while boosting their photoluminescence quantum yield from 6.05% in aqueous solution to 22.83% in the gel, primarily due to spatial confinement imposed by the polymer network. These solvent‐responsive structural dynamics directly regulate the photoluminescence output of the NCs, establishing a unified mechanism that couples actuation with optical signaling. The emission behavior is further tunable through cluster size variation, underscoring the modularity of the platform. Kinetic analysis reveals a two‐stage deswelling process, comprising an initial surface diffusion phase followed by subsequent network rearrangement, both of which correlate with real‐time luminescence evolution. This synchronized dual‐response capability is visually demonstrated in a biomimetic lotus‐shaped construct and validated in a leakage suppression scenario, where the gel rapidly (< 5 s) conforms to and seals damaged pipeline sites while emitting bright luminescence for immediate visual feedback. Together, these findings establish a minimalistic yet multifunctional nanocluster–gel platform that unifies photophysics and actuation under mild stimuli, offering a generalizable strategy for adaptive, self‐reporting materials in sensing, soft robotics, and environmental interfaces.

## Conflicts of Interest

The authors declare no conflicts of interest.

## Supporting information




**Supporting File 1**: adma73386‐sup‐0001‐SuppMat.docx.

**Supporting File 2**: adma73386‐sup‐0002‐Video.zip.

## Data Availability

The data that supports the findings of this study are available in the supplementary material of this article.

## References

[1] L.‐W. Xia , R. Xie , X.‐J. Ju , W. Wang , Q. Chen , and L.‐Y. Chu , “Nano‐Structured Smart Hydrogels with Rapid Response and High Elasticity,” Nature Communications 4 (2013): 2226, 10.1038/ncomms3226. PMC3731657 23900497

[2] K. Lei , Z. Li , D. Zhu , et al., “Polysaccharide‐Based Recoverable Double‐Network Hydrogel with High Strength and Self‐Healing Properties,” Journal of Materials Chemistry B 8 (2020): 794–802, 10.1039/C9TB01679A. 31904754

[3] Z. Li , P. Liu , X. Ji , et al., “Bioinspired Simultaneous Changes in Fluorescence Color, Brightness, and Shape of Hydrogels Enabled by AIEgens,” Advanced Materials 32 (2020): 1906493, 10.1002/adma.201906493. 32022969

[4] C. Li , A. Iscen , H. Sai , et al., “Supramolecular–covalent Hybrid Polymers for Light‐Activated Mechanical Actuation,” Nature Materials 19 (2020): 900–909, 10.1038/s41563-020-0707-7. 32572204

[5] S. Wu , H. Shi , W. Lu , et al., “Aggregation‐Induced Emissive Carbon Dots Gels for Octopus‐Inspired Shape/Color Synergistically Adjustable Actuators,” Angewandte Chemie International Edition 60 (2021): 21890–21898, 10.1002/anie.202107281. 34312961

[6] J. Wu , Y. Wang , P. Jiang , X. Wang , X. Jia , and F. Zhou , “Multiple Hydrogen‐Bonding Induced Nonconventional Red Fluorescence Emission in Hydrogels,” Nature Communications 15 (2024): 3482, 10.1038/s41467-024-47880-7. PMC11045767 38664408

[7] A. Cangialosi , C. Yoon , J. Liu , et al., “DNA Sequence–directed Shape Change of Photopatterned Hydrogels via High‐Degree Swelling,” Science 357 (2017): 1126–1130, 10.1126/science.aan3925. 28912239

[8] S. Wei , W. Lu , X. Le , et al., “Bioinspired Synergistic Fluorescence‐Color‐Switchable Polymeric Hydrogel Actuators,” Angewandte Chemie 131 (2019): 16389–16397, 10.1002/ange.201908437. 31475456

[9] B. Wu , M. Si , L. Hua , et al., “Cephalopod‐Inspired Chemical‐Gated Hydrogel Actuation Systems for Information 3D‐Encoding Display,” Advanced Materials 36 (2024): 2401659, 10.1002/adma.202401659. 38533903

[10] C. Ma , W. Lu , X. Yang , et al., “Bioinspired Anisotropic Hydrogel Actuators with On–Off Switchable and Color‐Tunable Fluorescence Behaviors,” Advanced Functional Materials 28 (2018): 1704568, 10.1002/adfm.201704568.

[11] B. Y. Wu , X. X. Le , Y. K. Jian , et al., “pH and Thermo Dual‐Responsive Fluorescent Hydrogel Actuator,” Macromolecular Rapid Communications 40 (2019): 1800648, 10.1002/marc.201800648. 30485580

[12] W. Lu , S. Wei , H. Shi , X. Le , G. Yin , and T. Chen , “Progress in Aggregation‐Induced Emission‐Active Fluorescent Polymeric Hydrogels,” Aggregate 2, no. 3 (2021): 37.

[13] C. Li , A. Iscen , L. C. Palmer , G. C. Schatz , and S. I. Stupp , “Light‐Driven Expansion of Spiropyran Hydrogels,” Journal of the American Chemical Society 142 (2020): 8447–8453, 10.1021/jacs.0c02201. 32330027

[14] M. Du and C. Li , “Engineering Supramolecular Hydrogels via Reversible Photoswitching of Cucurbit[8]Uril‐Spiropyran Complexation Stoichiometry,” Advanced Materials 36 (2024): 2408484, 10.1002/adma.202408484. 39188206

[15] D. Xia , P. Wang , X. Ji , N. M. Khashab , J. L. Sessler , and F. Huang , “Functional Supramolecular Polymeric Networks: the Marriage of Covalent Polymers and Macrocycle‐Based Host–Guest Interactions,” Chemical Reviews 120 (2020): 6070–6123, 10.1021/acs.chemrev.9b00839. 32426970

[16] X. Ji , M. Ahmed , L. Long , N. M. Khashab , F. Huang , and J. L. Sessler , “Adhesive Supramolecular Polymeric Materials Constructed from Macrocycle‐Based Host–guest Interactions,” Chemical Society Reviews 48 (2019): 2682–2697, 10.1039/C8CS00955D. 31012443

[17] X. Yang , M. Du , Z. Chu , and C. Li , “Synchronizing Multicolor Changes and Shape Deformation into Structurally Homogeneous Hydrogels via a Single Photochromophore,” Advanced Materials 37 (2025): 2500857, 10.1002/adma.202500857. 40059611

[18] S. Wei , Z. Li , W. Lu , et al., “Multicolor Fluorescent Polymeric Hydrogels,” Angewandte Chemie International Edition 60 (2021): 8608–8624, 10.1002/anie.202007506. 32864843

[19] S. L. Banerjee , K. Bhattacharya , S. Samanta , and N. K. Singha , “Self‐Healable Antifouling Zwitterionic Hydrogel Based on Synergistic Phototriggered Dynamic Disulfide Metathesis Reaction and Ionic Interaction,” ACS Applied Materials & Interfaces 10 (2018): 27391–27406, 10.1021/acsami.8b10446. 30084628

[20] Y. Amamoto , J. Kamada , H. Otsuka , A. Takahara , and K. Matyjaszewski , “Repeatable Photoinduced Self‐Healing of Covalently Cross‐Linked Polymers through Reshuffling of Trithiocarbonate Units,” Angewandte Chemie International Edition 50 (2011): 1660–1663, 10.1002/anie.201003888. 21308927

[21] H. Li , T. Wang , J. Han , et al., “Fluorescence Resonance Energy Transfer in Atomically Precise Metal Nanoclusters by Cocrystallization‐Induced Spatial Confinement,” Nature Communications 15 (2024): 5351, 10.1038/s41467-024-49735-7. PMC11196639 38914548

[22] Y.‐N. Yang , Q.‐Y. Wan , M.‐J. Zhu , et al., “Pressure‐Activated Efficient Near‐Infrared Luminescence in Atomically Precise Gold Nanoclusters,” Journal of the American Chemical Society 147 (2025): 26991–26999, 10.1021/jacs.5c09304. 40680210

[23] W.‐Q. Shi , L. Zeng , R.‐L. He , et al., “Near‐Unity NIR Phosphorescent Quantum Yield from a Room‐Temperature Solvated Metal Nanocluster,” Science 383 (2024): 326–330, 10.1126/science.adk6628. 38236955

[24] C. P. Joshi , M. S. Bootharaju , M. J. Alhilaly , and O. M. Bakr , “[Ag_25_(SR)_18_]^−^: The “Golden” Silver Nanoparticle,” Journal of the American Chemical Society 137 (2015): 11578–11581, 10.1021/jacs.5b07088. 26322865

[25] Q. Li , M. Zhou , W. Y. So , et al., “A Mono‐Cuboctahedral Series of Gold Nanoclusters: Photoluminescence Origin, Large Enhancement, Wide Tunability, and Structure–Property Correlation,” Journal of the American Chemical Society 141 (2019): 5314–5325, 10.1021/jacs.8b13558. 30860834

[26] B. Zhang , J. Chen , Y. Cao , O. J. H. Chai , and J. Xie , “Ligand Design in ligand‐Protected Gold Nanoclusters,” Small 17, no. 27 (2021): 2004381. 10.1002/smll.202004381 33511773

[27] Z. Gan , Y. Lin , L. Luo , et al., “Fluorescent Gold Nanoclusters with Interlocked Staples and a Fully Thiolate‐Bound Kernel,” Angewandte Chemie International Edition 55 (2016): 11567–11571, 10.1002/anie.201606661. 27529838

[28] Z. Luo , X. Yuan , Y. Yu , et al., “From Aggregation‐Induced Emission of Au(I)–Thiolate Complexes to Ultrabright Au(0)@Au(I)–Thiolate Core–Shell Nanoclusters,” Journal of the American Chemical Society 134 (2012): 16662–16670, 10.1021/ja306199p. 22998450

[29] M. Sugiuchi , J. Maeba , N. Okubo , M. Iwamura , K. Nozaki , and K. Konishi , “Aggregation‐Induced Fluorescence‐to‐Phosphorescence Switching of Molecular Gold Clusters,” Journal of the American Chemical Society 139 (2017): 17731–17734, 10.1021/jacs.7b10201. 29178782

[30] Y. Xiao , Z. Wu , Q. Yao , and J. Xie , “Luminescent Metal Nanoclusters: Biosensing Strategies and Bioimaging Applications,” Aggregate 2 (2021): 114–132, 10.1002/agt2.11.

[31] X. Luo , J. Kong , H. Xiao , et al., “Noncovalent Interaction Guided Precise Photoluminescence Regulation of Gold Nanoclusters in both Isolate Species and Aggregate States,” Angewandte Chemie International Edition 63, no. 27 (2024): 202404129. 10.1002/anie.202404129 38651974

[32] H. Deng , K. Huang , L. Xiu , et al., “Bis‐Schiff Base Linkage‐Triggered Highly Bright Luminescence of Gold Nanoclusters in Aqueous Solution at the Single‐Cluster Level,” Nature Communications 13 (2022): 3381, 10.1038/s41467-022-30760-3. PMC9192726 35697695

[33] W. Dong , L. Hong , Y. Yang , et al., “Softening of Electron‐Acoustic Phonon Coupling via Core Symmetry Breaking in Metal Nanoclusters,” Laser & Photonics Reviews 0 (2026): 02646, 10.1002/lpor.202502646.

[34] Z. Wu , Q. Yao , O. J. H. Chai , et al., “Unraveling the Impact of Gold(I)–Thiolate Motifs on the Aggregation‐Induced Emission of Gold Nanoclusters,” Angewandte Chemie 132 (2020): 10020–10025, 10.1002/ange.201916675. 32011796

[35] J. Luo , Z. Xie , J. W. Lam , et al., “Aggregation‐Induced Emission of 1‐Methyl‐1,2,3,4,5‐Pentaphenylsilole,” Chemical Communications 18 (2001): 1740–1741, 10.1039/b105159h. 12240292

[36] J. Mei , N. L. Leung , R. T. Kwok , J. W. Lam , and B. Z. Tang , “Aggregation‐Induced Emission: Together We Shine, United We Soar!,” Chemical Reviews 115 (2015): 11718–11940, 10.1021/acs.chemrev.5b00263. 26492387

[37] K. Pyo , V. D. Thanthirige , K. Kwak , P. Pandurangan , G. Ramakrishna , and D. Lee , “Ultrabright Luminescence from Gold Nanoclusters: Rigidifying the Au(I)–Thiolate Shell,” Journal of the American Chemical Society 137 (2015): 8244–8250, 10.1021/jacs.5b04210. 26061198

[38] Z. Liu , Y. Li , E. Kahng , et al., “Tailoring the Electron–Phonon Interaction in Au_25_(SR)_18_ Nanoclusters via Ligand Engineering and Insight into Luminescence,” ACS Nano 16 (2022): 18448–18458, 10.1021/acsnano.2c06586. 36252530

[39] Y. Bi , Z. Wang , T. Liu , et al., “Supramolecular Chirality from Hierarchical Self‐Assembly of Atomically Precise Silver Nanoclusters Induced by Secondary Metal Coordination,” ACS Nano 15 (2021): 15910–15919, 10.1021/acsnano.1c03824. 34542271

[40] J. Shen , J. Fu , P. Mahato , et al., “Gold Nanocluster Isomerization Drives Supramolecular Transition from Heterochiral to Homochiral Helices,” ACS Nano 19 (2025): 31516–31526, 10.1021/acsnano.5c08207. 40844432

[41] Z. Xie , P. Sun , Z. Wang , et al., “Metal–Organic Gels from Silver Nanoclusters with Aggregation‐Induced Emission and Fluorescence‐to‐Phosphorescence Switching,” Angewandte Chemie International Edition 59 (2020): 9922–9927, 10.1002/anie.201912201. 31573132

[42] Z. Luo , K. Zheng , and J. Xie , “Engineering Ultrasmall Water‐Soluble Gold and Silver Nanoclusters for Biomedical Applications,” Chemical Communications 50 (2014): 5143–5155, 10.1039/C3CC47512C. 24266029

[43] S. Li , J. Wei , Q. Yao , X. Song , J. Xie , and H. Yang , “Emerging Ultrasmall Luminescent Nanoprobes for in Vivo Bioimaging,” Chemical Society Reviews 52 (2023): 1672–1696, 10.1039/D2CS00497F. 36779305

[44] F. Wang , Y. Zhong , O. Bruns , Y. Liang , and H. Dai , “In Vivo NIR‐II Fluorescence Imaging for Biology and Medicine,” Nature Photonics 18 (2024): 535–547, 10.1038/s41566-024-01391-5.

[45] T. Lindvig , M. L. Michelsen , and G. M. Kontogeorgis , “A Flory–Huggins Model Based on the Hansen Solubility Parameters,” Fluid Phase Equilibria 203 (2002): 247–260, 10.1016/S0378-3812(02)00184-X.

[46] R. A. Orwoll , “The Polymer‐Solvent Interaction Parameter X,” Rubber chemistry and technology 50 (1977): 451–479, 10.5254/1.3535155.

[47] Y. Yu , X. Chen , Q. Yao , Y. Yu , N. Yan , and J. Xie , “Scalable and Precise Synthesis of Thiolated Au_10–12_, Au_15_, Au_18_, and Au_25_ Nanoclusters via pH Controlled CO Reduction,” Chemistry of Materials 25 (2013): 946–952, 10.1021/cm304098x.

[48] O. Yanshyna , M. J. Białek , O. V. Chashchikhin , and R. Klajn , “Encapsulation within a Coordination Cage Modulates the Reactivity of Redox‐Active Dyes,” Communications Chemistry 5, no. 1 (2022): 44. 36697669 10.1038/s42004-022-00658-8 PMC9814915

[49] I. Coropceanu , E. M. Janke , J. Portner , et al., “Self‐Assembly of Nanocrystals into Strongly Electronically Coupled All‐Inorganic Supercrystals,” Science 375 (2022): 1422–1426, 10.1126/science.abm6753. 35324292

[50] M. Liu , L. Fang , Y. Li , M. Gong , A. Xu , and Z. Deng , ““Flash” Preparation of Strongly Coupled Metal Nanoparticle Clusters with Sub‐nm Gaps by Ag + Soldering: Toward Effective Plasmonic Tuning of Solution‐Assembled Nanomaterials,” Chemical Science 7 (2016): 5435–5440, 10.1039/C6SC01407K. 30034682 PMC6021751

[51] T. Chen , S. Yang , J. Chai , et al., “Crystallization‐Induced Emission Enhancement: a Novel Fluorescent Au‐Ag Bimetallic Nanocluster with Precise Atomic Structure,” Science Advances 3, no. 8 (2017): 1700956. 10.1126/sciadv.1700956 PMC5562423 28835926

[52] Z. Wu , Q. Yao , S.‐Q. Zang , and J. Xie , “Aggregation‐Induced Emission in Luminescent Metal Nanoclusters,” National Science Review 8 (2021): nwaa208, 10.1093/nsr/nwaa208. 34691661 PMC8288168

[53] D. G. Archer and P. Wang , “The Dielectric Constant of Water and Debye‐Hückel Limiting Law Slopes,” Journal of Physical and Chemical Reference Data 19 (1990): 371–411, 10.1063/1.555853.

[54] F. Hassion and R. Cole , “Dielectric Properties of Liquid Ethanol and 2‐Propanol,” The Journal of Chemical Physics 23 (1955): 1756–1761, 10.1063/1.1740575.

[55] S. Maity , S. Kolay , S. Chakraborty , A. Devi , and A. Patra , “A Comprehensive Review of Atomically Precise Metal Nanoclusters with Emergent Photophysical Properties towards Diverse Applications,” Chemical Society Reviews 54 (2025): 1785–1844, 10.1039/D4CS00962B. 39670813

[56] Z. Liu , L. Luo , and R. Jin , “Visible to NIR‐II Photoluminescence of Atomically Precise Gold Nanoclusters,” Advanced Materials 36 (2024): 2309073, 10.1002/adma.202309073. 37922431

[57] H. Lin , Y. Cao , Q. Yao , T. Chen , H. Shan , and J. Xie , “The Synthesis of Fluorescent Nanoclusters Based on the Etching Reaction,” Nano Research 17 (2024): 4631–4638, 10.1007/s12274-023-6391-6.

[58] J. Shen , Z. Wang , D. Sun , et al., “Self‐Assembly of Water‐Soluble Silver Nanoclusters: Superstructure Formation and Morphological Evolution,” Nanoscale 9 (2017): 19191–19200, 10.1039/C7NR06359H. 29186220

[59] W. Feng and Z. Wang , “Tailoring the Swelling‐Shrinkable Behavior of Hydrogels for Biomedical Applications,” Advanced Science 10 (2023): 2303326, 10.1002/advs.202303326. 37544909 PMC10558674

[60] Y. Alsaid , S. Wu , D. Wu , et al., “Tunable Sponge‐like Hierarchically Porous Hydrogels with Simultaneously Enhanced Diffusivity and Mechanical Properties,” Advanced Materials 33, no. 20 (2021): 2008235. 10.1002/adma.202008235 33829563

[61] J. R. Quintana , N. E. Valderruten , and I. Katime , “Synthesis and Swelling Kinetics of Poly(Dimethylaminoethyl acrylate methyl chloride quaternary‐ co ‐Itaconic acid) Hydrogels,” Langmuir 15 (1999): 4728–4730, 10.1021/la980982+.

[62] F. Ganji , F. S. Vasheghani , and F. E. Vasheghani , “Vasheghani‐Fara Hani E,” Iranian Polymer Journal 19 (2010): 375.

[63] H. Omidian , S. Hashemi , P. Sammes , and I. Meldrum , “A Model for the Swelling of Superabsorbent Polymers,” Polymer 39 (1998): 6697–6704, 10.1016/S0032-3861(98)00095-0.

[64] N. Yavari and S. Azizian , “Mixed Diffusion and Relaxation Kinetics Model for Hydrogels Swelling,” Journal of Molecular Liquids 363 (2022): 119861, 10.1016/j.molliq.2022.119861.

[65] T. Jayaramudu , H.‐U. Ko , H. C. Kim , J. W. Kim , and J. Kim , “Swelling Behavior of Polyacrylamide–Cellulose Nanocrystal Hydrogels: Swelling Kinetics, Temperature, and pH Effects,” Materials 12 (2019): 2080, 10.3390/ma12132080. 31261618 PMC6650916

[66] A. L. Daniel‐da‐Silva , J. Moreira , R. Neto , A. C. Estrada , A. M. Gil , and T. Trindade , “Impact of Magnetic Nanofillers in the Swelling and Release Properties of κ‐Carrageenan Hydrogel Nanocomposites,” Carbohydrate Polymers 87 (2012): 328–335, 10.1016/j.carbpol.2011.07.051. 34662970

[67] L. Serra , J. Doménech , and N. A. Peppas , “Drug Transport Mechanisms and Release Kinetics from Molecularly Designed Poly(acrylic acid‐g‐Ethylene glycol) Hydrogels,” Biomaterials 27 (2006): 5440–5451, 10.1016/j.biomaterials.2006.06.011. 16828864

[68] Y. Li , X. Li , C. Chen , et al., “A Rapid, Non‐Invasive and Non‐Destructive Method for Studying Swelling Behavior and Microstructure Variations of Hydrogels,” Carbohydrate Polymers 151 (2016): 1251–1260, 10.1016/j.carbpol.2016.06.054. 27474678

[69] K. László , A. Fluerasu , A. Moussaïd , and E. Geissler , “Deswelling Kinetics of PNIPA Gels,” Soft Matter 6, no. 18 (2010): 4335–4338.

[70] S. Boral , A. Saxena , and H. Bohidar , “Syneresis in Agar Hydrogels,” International Journal of Biological Macromolecules 46 (2010): 232–236, 10.1016/j.ijbiomac.2009.12.008. 20035783

