# Supplementary material for: Synchronizing Tunable Luminescence and Shape Morphing in a Metal Nanocluster–Enabled Hydrogel Platform
Source: Adv Mater. 2026 May 14;38(34):e73386. doi: 10.1002/adma.73386 (PMC13274691; doi:10.1002/adma.73386)

Supporting Information

Synchronizing Tunable Luminescence and Shape Morphing in a Metal Nanocluster–Driven Hydrogel Platform

*Hongbin Lin^#^, Zening Huang^#^, Yongshi He, Hongda Wei, Zhucheng Yang, Zhiqiang Hu, Yang Zhou, Xiaorong Song*, Sanyang Han*, and Jianping Xie**

^#^These authors contributed equally to this work.

H. Lin, H. Wei, Z. Hu, Y. Zhou, Prof. Han

Institute of Biopharmaceutical and Health Engineering,

Tsinghua Shenzhen International Graduate School (SIGS), Tsinghua University

Shenzhen, China, 518055

E-mail: [hansanyang@sz.tsinghua.edu.cn](mailto:hansanyang@sz.tsinghua.edu.cn)

H. Lin, Z. Yang, Prof. J. Xie

Department of Chemical and Biomolecular Engineering
National University of Singapore
4 Engineering Drive 4, Singapore 117585, Singapore
E-mail: [chexiej@nus.edu.sg](mailto:chexiej@nus.edu.sg)

Prof. Han

Key Laboratory of Industrial Biocatalysis

Ministry of Education, Tsinghua University

Beijing, China, 100084

Z. Huang

Department of Gastric Surgery
Fujian Medical University Union Hospital,
Fuzhou, China, 350001

Y. He, Prof. X. Song

MOE Key Laboratory for Analytical Science of Food Safety and Biology and State Key Laboratory of Photocatalysis on Energy and Environment

College of Chemistry, Fuzhou University

Fuzhou, China, 350108

E-mail: [xrsong@fzu.edu.cn](mailto:xrsong@fzu.edu.cn)

**Experimental Section**

***Chemical and Materials.*** Glutathione (GSH), 6-mercaptopropionic acid (MHA), 3-mercaptopropionic acid (MPA), 2-mercaptonicotinic acid (H₂mna), silver nitrate (AgNO_3_, ≥ 99.9% trace metals basis), tetrachloroauric(III) acid (HAuCl_4_·3H_2_O, ≥ 99.99% metals basis), and sodium hydroxide (≥ 99.8%), acrylamide, *N,N′*-methylenebisacrylamide were all bought from Sigma-Aldrich. And ethanol was purchased from Fisher. The *N,N,N',N'*-tetramethyl ethylenediamine (TEMED), ammonium persulfate (APS), resolving gel buffer (1.5 M Tris-HCl buffer, pH = 8.8), 40% acrylamide/bis solution (19:1) were purchased from Bio-Rad. Carbon monoxide (CO, 99.9%) was acquired from Singapore Oxygen Air Liquide Pte Ltd (SOXAL). All used chemicals did not need the further purification. Ultrapure Millipore water (18.2 MΩ) was used in all experiments. All the glassware ware washed by Aqua Regia before using.

***Synthesis of*** ***Au_10-12_, Au_15_, and Au_18_ NCs protected by GSH.*** In a typical synthesis of Au_10-12_(GSH)_10-12_ NCs, GSH solution (1.2 ml, 50 mM) and HAuCl_4_ (1.5 ml, 20 mM) were added into a 40-ml glass flask containing 27.3 ml ultrapure water. After vigorously stirring 2 minutes at 500 rpm under room temperature, the pH of the reaction solution was tuned to ~7.0 via drop-wise addition of 1M aqueous NaOH solution (~ 0.18 ml). Then, 1 bar CO was bubbled into the reaction solution. After 2 minutes, the sealed airtight reaction mixture was allowed to proceed for 24 hours stirring at 500 rpm under room temperature to obtain Au_10-12_(GSH)_10-12_ NCs. The synthesis processes for Au_15_(GSH)_13_ and Au_18_(GSH)_14_ were similar to that of Au_10-12_(GSH)_10-12_ NCs except the pH value of reaction solution. In the synthesis of Au_15_(GSH)_13_, the added amount of 1M aqueous NaOH solution was ~ 0.195 ml to control the pH value of reaction solution at 9.0. As for Au_18_(GSH)_14_, the pH value of reaction solution was controlled at 10.0 by adding ~ 0.435 ml 1M aqueous NaOH solution. Except the differences in pH value, other procedures for the synthesis of Au_10-12_, Au_15_, and Au_18_ NCs are the same.

***Synthesis of Au_18_ NCs protected by MPA*.** In a typical synthesis, MPA (1.2 mL, 50 mM) and HAuCl₄ (1.5 mL, 20 mM) were mixed with 27.3 mL ultrapure water under stirring (500 rpm). After 2 min, the pH was adjusted to ~11.0 using 1 M NaOH. CO (1 bar) was bubbled for 2 min, and the sealed reaction was maintained at room temperature for 24 h to yield Au₁₈(MPA)₁₄ NCs.

***Synthesis of Au_22_ NCs protected by MHA.*** Au₂₂(MHA)₁₈ NCs were prepared via an etching reaction using Au₂₅(MHA)₁₈ as the precursor. Au₂₅(MHA)₁₈ was first synthesized by mixing MHA (8 mM, 4 mL) and HAuCl₄ (50 mM, 0.2 mL) in ultrapure water (5.8 mL), followed by adjusting the pH to 12.0 with 1 M NaOH, bubbling CO for 2 min, and reacting at room temperature for 24 h. The crude product was purified by ultrafiltration using a 5000 Da molecular weight cutoff membrane. The purified Au₂₅(MHA)₁₈ solution was then concentrated to about 1 mL, diluted to give an absorbance of 0.35–0.45 at 680 nm, and the Au_25_ NCs concentration was determined by inductively coupled plasma optical emission spectroscopy (ICP-OES). The solution was subsequently mixed with excess free MHA (5 mM) at a thiol-to-Au ratio of 2.0. After adjusting the pH to 10.2–10.5 with 1 M NaOH, the mixture was stirred in air at room temperature (500 rpm) for 5 days to induce etching and form Au₂₂(MHA)₁₈ NCs.

***Synthesis of Ag_6_ NCs protected by Hmna.*** A mixture of AgNO₃ (167 mg, 1 mmol) and H₂mna (155 mg, 1 mmol) was dispersed in water (6 mL) in a conical flask and subjected to ultrasonic treatment (160 W, 40 kHz) for 20 min at room temperature. Subsequently, an aqueous NH₃ solution (25%, 0.5 mL) was added dropwise, leading to the dissolution of the precipitate and the formation of a clear yellow solution. The resulting solution was directly used for subsequent usage.

***Fabrication of NC-based gels.*** To prepare luminescent nanocluster-based films, first, the prepared MNCs solution (4.5 ml, ~0.023 mM) is added to a glass vial containing acrylamide (2.85 g) and *N,N'*-methylenebisacrylamide (0.15 g). The mixture is then subjected to ultrasonic treatment until completely combined and a transparent solution is achieved. Subsequently, 2.5 ml of resolving gel, 50 μL of APS, and 5 μL of TEMED are sequentially added to the mixture, stirring thoroughly after each addition to obtain an NC-based gel solution. The resulting NC-based gel solution is then poured into a Bio-Rad Mini-PROTEAN® Tetra Cell or PROTEAN® II xi Cell system. After approximately 30 minutes, the gel solidifies, resulting in a luminescent NC-based film.

***Characterizations******.*** Mettler-Toledo FE 20 pH-meter was used to record the solution pH. Fluorescence measurements were conducted in a LS-55 luminescence spectrometer from Perkin-Elmer. UV-vis spectra were obtained on a Shimadzu UV-1800 spectrometer. The morphologies of swollen and deswollen NC-based were taken by scanning electron microscope (SEM, Apreo 2S HiVac). The fluorescent images were taken on TFML-30 2UV transilluminator from analytikjena. Confocal images were captured by a laser-scanning confocal microscopy system (NIKON, Al, Japan). Inverted fluorescence image was taken on a Nikon Eclipse Ti-S inverted microscope. Dynamic light scattering (DLS) was performed on a Zetasizer Nano ZS instrument (Malvern, England).

***Experimental methods of ^1^H NMR measurements.*** To perform the ^1^H NMR measurements of the polyacrylamide gel monomer solution, acrylamide (2.85 g) and *N,N′*-methylenebisacrylamide (0.15 g) were first dissolved in D_2_O (4.5 ml), followed by the addition of resolving gel buffer (2.5 ml). The solution was thoroughly mixed and transferred into an NMR tube, and the ^1^H NMR measurements were conducted prior to gelation. As for the ¹H NMR measurements of the Au₁₈-based system, a concentrated Au₁₈ solution was first diluted with D₂O to give a NC concentration of approximately 0.023 mM. An aliquot of this solution was directly used for the ¹H NMR measurement of Au₁₈ in D₂O, while the remaining 4.5 mL was used as the solvent to prepare the gel monomer solution following the same procedure described above. All ¹H NMR spectra were measured on a Bruker AVANCE 400 spectrometer operating at 400 MHz using D₂O as the solvent.

**The swelling and deswelling dynamics fitting of NC-based gel.**

The amount of water retained in the NC-based gel can be expressed mathematically in different ways as a swelling ratio. The equilibrium swelling ratio ($W_{\infty}$) of the gel can be calculated using Eq. (1).

$W_{\infty}=\frac{M_{\infty}-M_{0}}{M_{0}}$ (1)

Where $M_{\infty}$ and $M_{0}$ are the weight of the equilibrium swollen gel and the weight of the equilibrium deswollen gel in anhydrous ethanol, respectively.

The isothermal swelling ratio at different time *t* is calculated based on Eq. (2)

$W_{t}=\frac{M_{t}-M_{0}}{M_{0}}$ (2)

Where $W_{t}$ and $M_{t}$ are the swelling ratio of the gel at time *t* and the weight of the gel at time *t*, respectively. The swelling kinetics can be characterized by fitting the experimental data to various models. The model that yields the highest correlation coefficient (R^2^) is considered the most accurate in describing the swelling behavior. A selection of the most commonly used models is provided below:

1. First-order kinetics: it can be expressed as:

$\frac{dW}{dt}=K(W_{\infty}-W_{t})$ (3)

$-\ln\left( 1-\frac{W_{t}}{W_{\infty}} \right)=Kt$ (4)

Where $(W_{\infty}-W_{t})$ represents the remaining swelling capacity, and *K* is the proportionality constant between the swelling rate and the remained swelling capacity.

1. Second-order kinetics: it can be expressed as:

$\frac{dW}{dt}=K(W_{\infty}-W_{t})^{2}$ (5)

$\frac{t}{W_{t}}=A+Bt$ (6)

Where *K* represents the second order swelling rate constant; *B* denotes the reciprocal of the equilibrium swelling ratio $W_{\infty}$, expressed as $B=1/{W_{\infty}}$; meanwhile, *A* represents the inverse of the hydrogel’s initial swelling rate, defined as $A=1/{({dW}/{dt)_{0}}}.$

The early-stage swelling data (${W_{t}}/{W_{\infty}}$≤ 60%) were fitted using Ritger-Peppas model:^[54-56]^

$\frac{W_{t}}{W_{\infty}}=kt^{n}$ (7)

$\ln\left( \frac{W_{t}}{W_{\infty}} \right)=nlnt+lnk$ (8)

Where *k* is a proportionality constant related to the network structure, and *n* characterizes the diffusion behavior of water molecules within the hydrogel matrix. For swellable hydrogel systems, when *n* < 0.5, the swelling follows a Fickian diffusion mechanism; When *n* = 0.5, the system exhibits ideal Fickian diffusion control. For Fickian swelling, *n* values between 0.45 and 0.5 are typically observed, where the rate of water diffusion is lower than the rate of polymer fiber relaxation. When 0.5 < *n* < 1, the swelling process is classified as non-Fickian transport, indicating a cooperative influence of solvent diffusion and polymer fiber relaxation, commonly due to slower fiber relaxation dynamics.

Regarding the deswelling behavior of NC-based gels, the deswelling curves can be analyzed by fitting the data to an exponential decay model, which can be expressed as:

$\frac{M_{t}-M_{\infty}}{M_{0}-M_{\infty}}=A_{1}e^{-k_{1}t}+A_{2}e^{-k_{2}t}$ (9)

Where ${(M_{t}-M_{\infty})}/{(M_{0}-M_{\infty})}$ represents the ratio of the change in the mass of the hydrogel ($M_{t}-M_{\infty}$) at time *t* to the total change in mass from the initial completely swelling state to the equilibrium state ($M_{0}-M_{\infty}$) during the deswelling process. This ratio indicates the normalized swelling degree of the NC-based gel at time *t*, showing the proportion of the gel's mass relative to both its initial mass and its maximum swelling capacity. $A_{1}$, $A_{2}$, $k_{1}$, and $k_{2}$ are fitting parameters, including pre-exponential factors and deswelling rate constants. And deswelling time constant ($\tau_{1}$, $\tau_{2}$) are corresponding to $1/{k_{1}}$ and $1/{k_{2}}$, respectively.

Supplementary Figures and Table


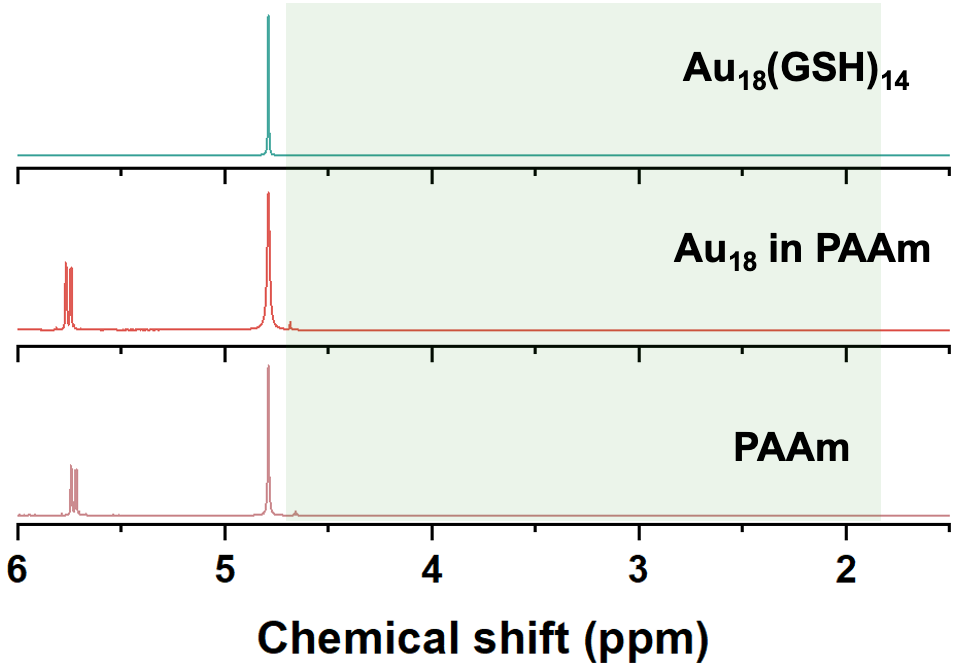


**Figure S1.** Full spectra of Partial enlarged ¹H NMR spectra of Au₁₈(GSH)_14_ in D_2_O (~0. 23 mM), Au₁₈ in polyacrylamide (PAAm) monomer solution, and Au₁₈-free PAAm monomer solution.


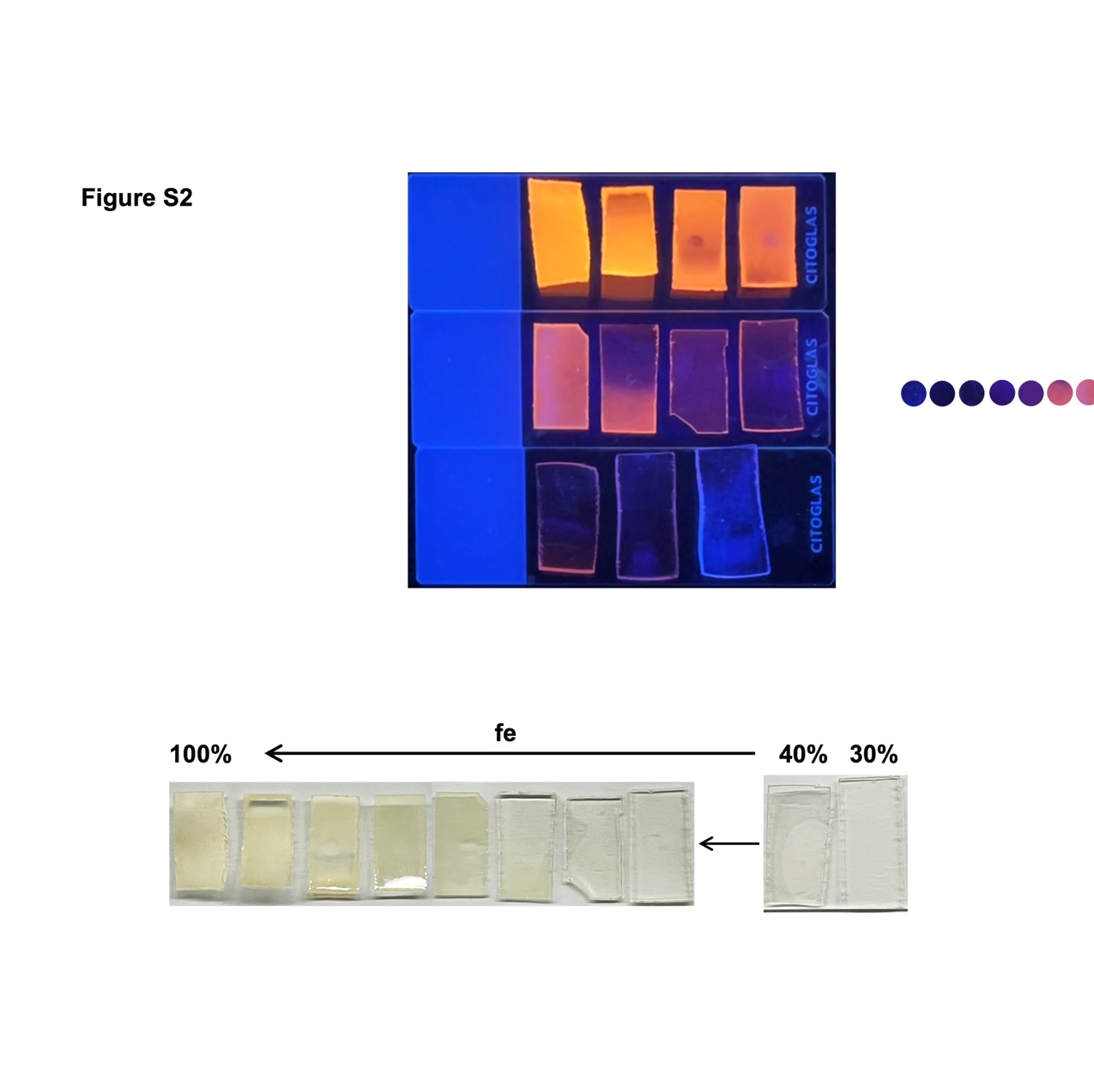


**Figure S2.** Photographs of gels in ethanol solutions with varying *fe*, showing increased volume shrinkage as *fe* increases from 30% to 100%.


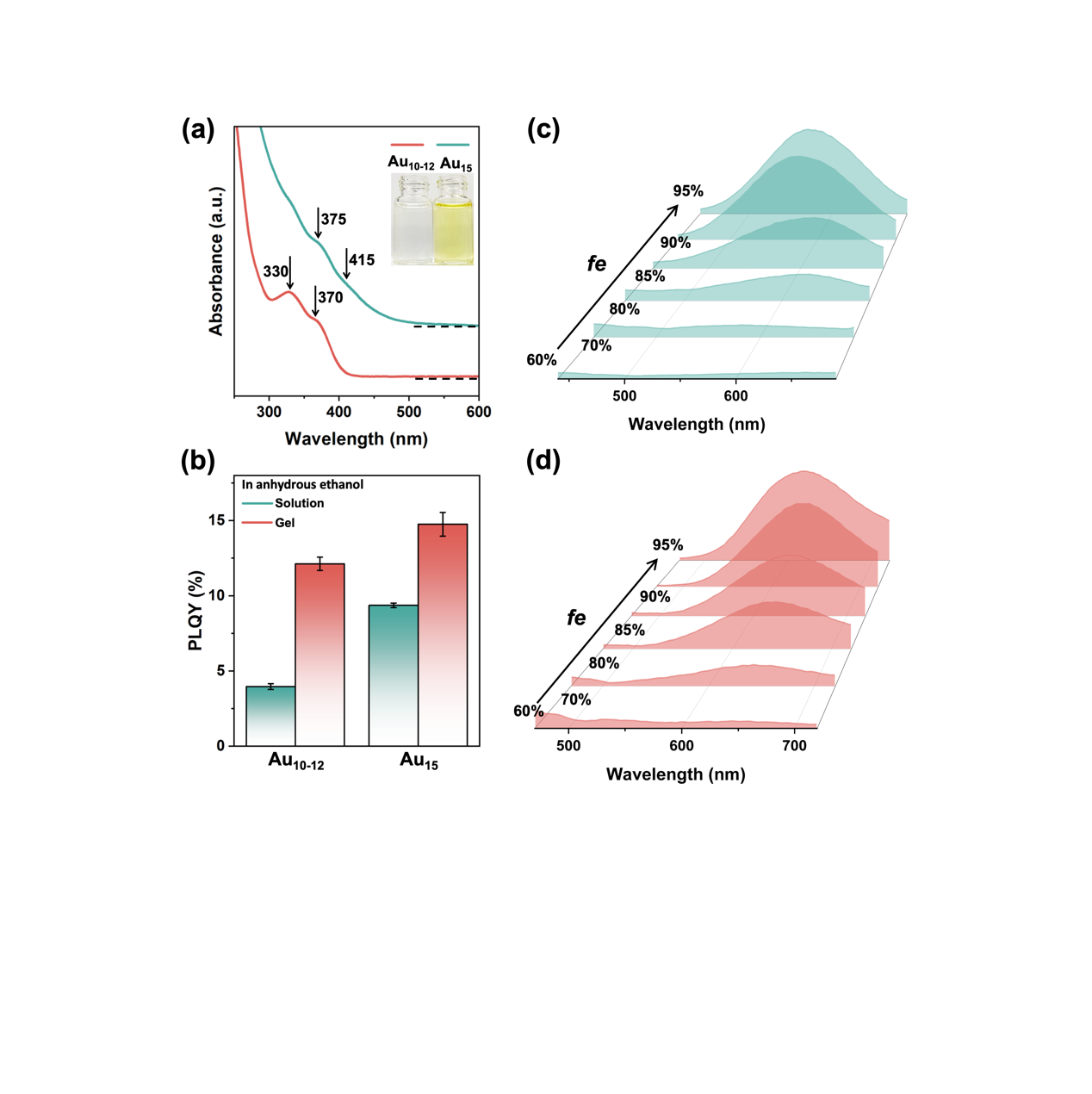


**Figure S3.** (a) UV-vis spectra of Au_10-12_ and Au_15_ NCs with corresponding digital photos (inset). (b) PLQY comparison between aggregated NCs in liquid phase and deswollen gels. Emission spectra of (c) Au_10-12_ NCs and (d) Au_15_ NCs under varying *fe* (λ_ex_ = 365 nm).

Besides Au_18_-based gels, gels prepared from other MNCs AIE properties also display similar behaviors, as shown in Figure S3. we first synthesized Au_10-12_ and Au_15_ MNCs by simply modifying the pH during the synthesis of the Au_18_ NCs, adjusting it to pH 7 and 9, respectively. The UV-vis absorption spectra (Figure S3a) show characteristic absorption peaks at 330 nm and 370 nm when the reaction pH is 7.0, and at 375 nm and 415 nm when the reaction pH is 9.0, which are completely consistent with previously reported results, confirming the successful synthesis of Au_10-12_ and Au_15_ NCs, respectively. By varying the *fe* value from 60% to 95%, the emission intensity of Au_10-12_ and Au_15_ NCs was improved significantly (Figure S3c and S3d), reflecting the remarkable AIE properties of both NC species. Subsequently, similar to the Au_18_-based gel, we prepared Au_10-12_-based gel and Au_15_-based gel using the same method. When immersed in ethanol solutions with varying *fe* value, the luminescent intensity of the gels similarly increased with the extent of deswelling, and the luminescence also followed a good exponential growth fit versus *fe* values (presented in the inset in Figure 2e (R^2^ = 0.991) and 2f (R^2^ = 0.995)). Compared to the PLQY of the aggregation states in the solution state (Au_10-12_: 3.96%, Au_15_:12.13%), the PLQY in nanocluster-gel system, after deswelling in anhydrous ethanol, was also significantly enhanced, especially reaching 9.3% and 14.73% for the Au_10-12_- and Au_15_- based gel, respectively (Figure S3b). These results collectively demonstrate that the luminescent performance of nanocluster-platform can be effectively tuned through solvent-induced deswelling and the control of aggregation behavior.

*
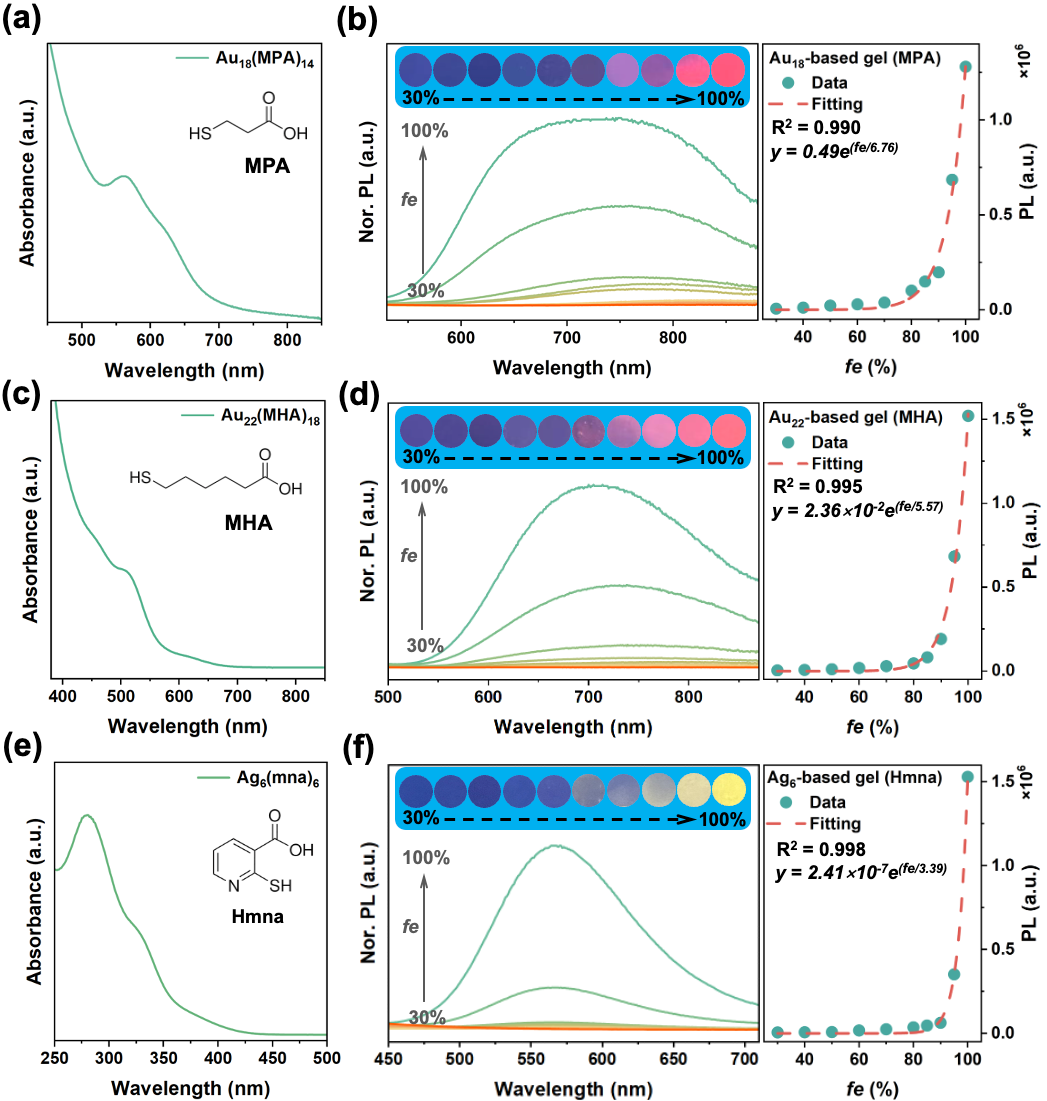
*

**Figure S4** (a, c, e) UV–vis absorption spectra of the as-synthesized Au₁₈(MPA)₁₄, Au₂₂(MHA)₁₈, and Ag₆(mna)₆ NCs, measured in water (a, c) and methanol (e). (b, d, f) Emission spectra of the corresponding nanocluster-based gels immersed in ethanol/water mixtures with ethanol volume fraction (*fₑ*) ranging from 30% to 100%, along with fluorescence images under UV illumination (top) and exponential fits of fluorescence intensity versus *fₑ*. All emission spectra were recorded with an excitation wavelength of 365 nm, consistent with the UV illumination used for imaging.


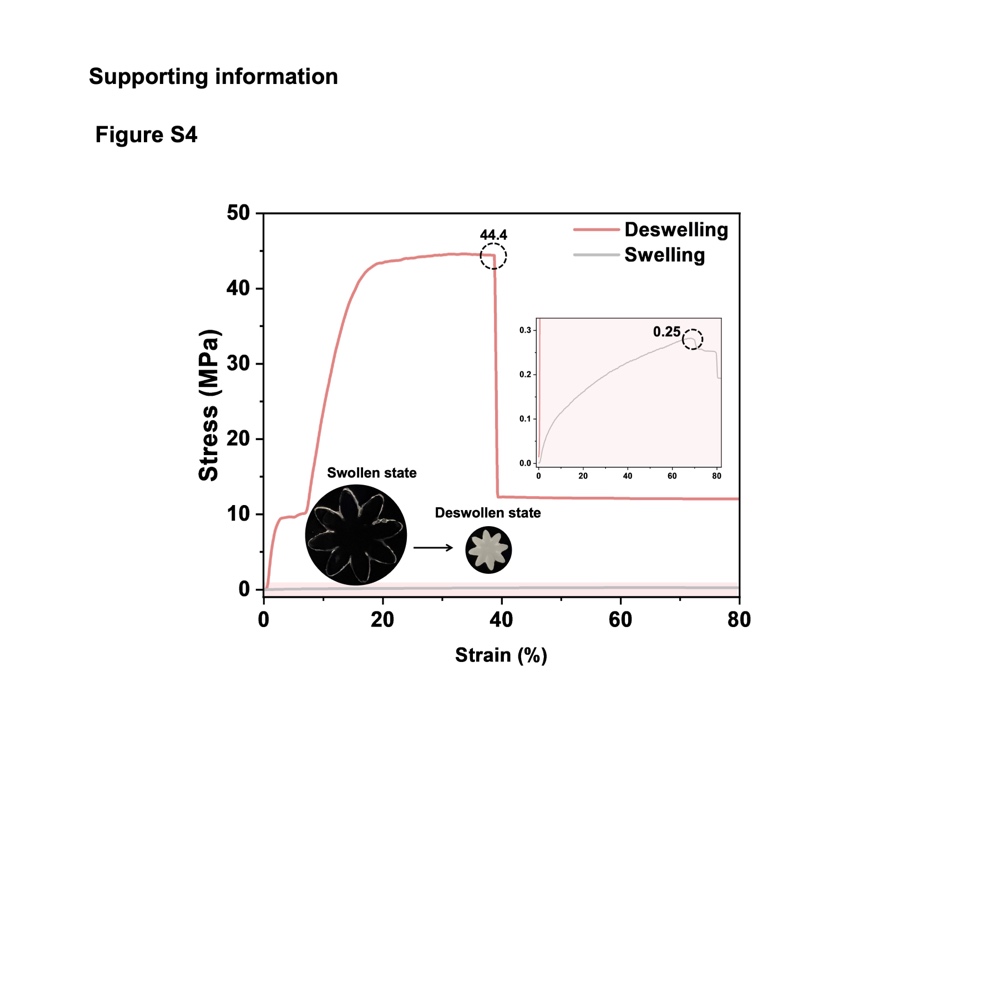


**Figure S5.** Comparison of tensile strength between swollen and deswollen states of the NC-based gel.


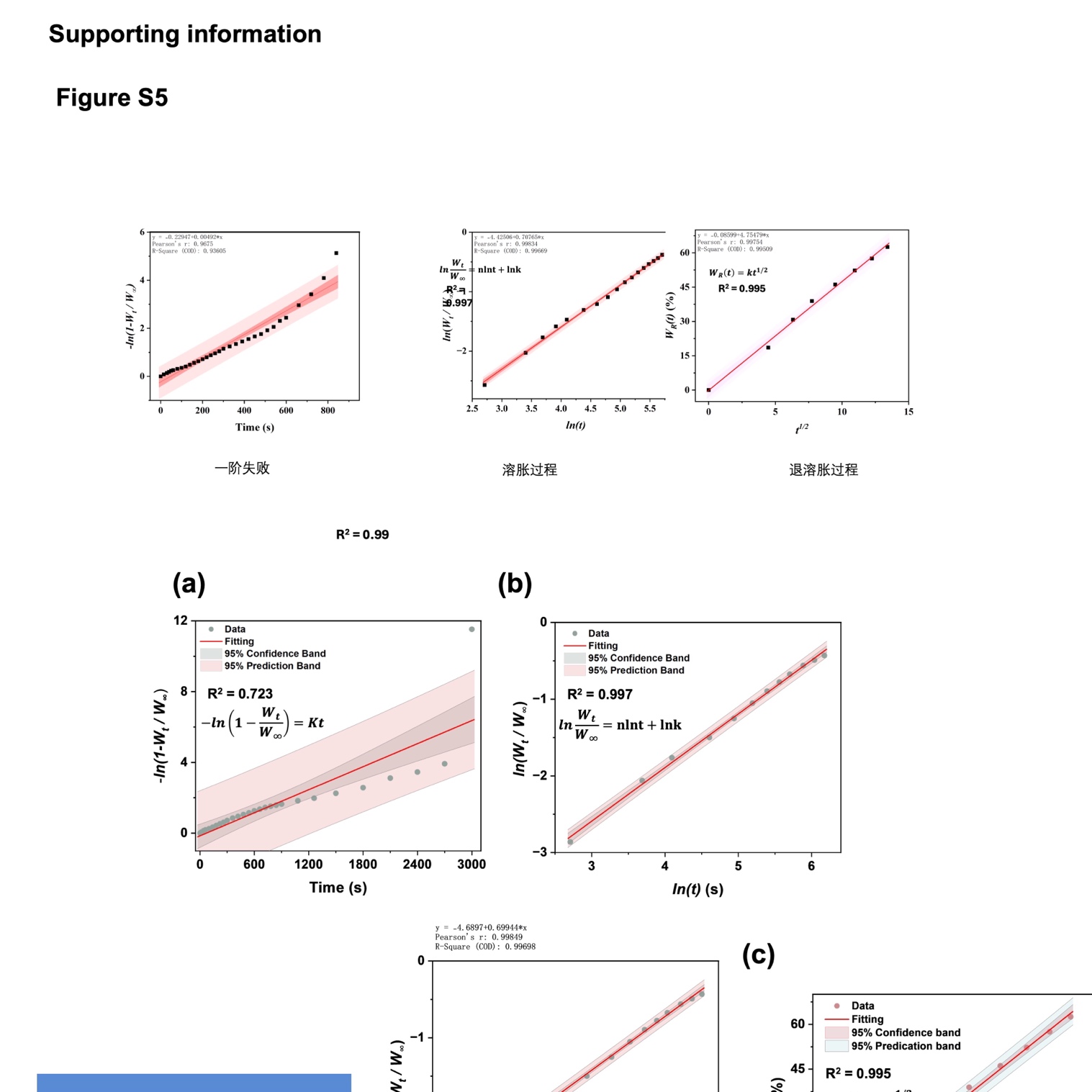


**Figure S6.** **(a)** First-order kinetic fitting of gel during the swelling process. **(b)** Fitting lines of diffusion mechanism of deswollen hydrogels in pure water.


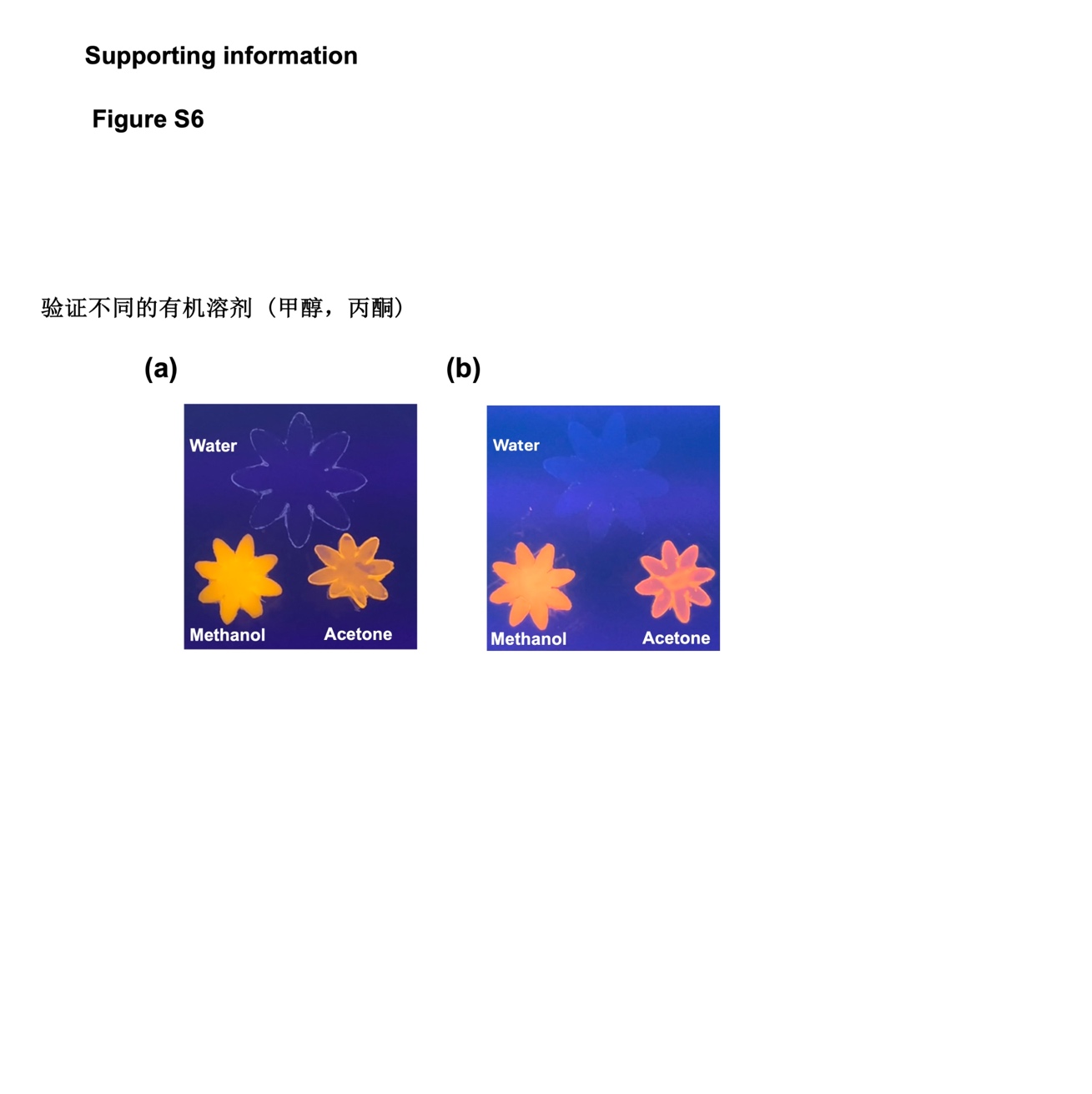


**Figure S7.** Fluorescence images of **(a)** Au_15_- and **(b)** Au_18_-based gels after deswelling in methanol and acetone, demonstrating the general organic solvent-responsive behavior of the cluster-based gels.


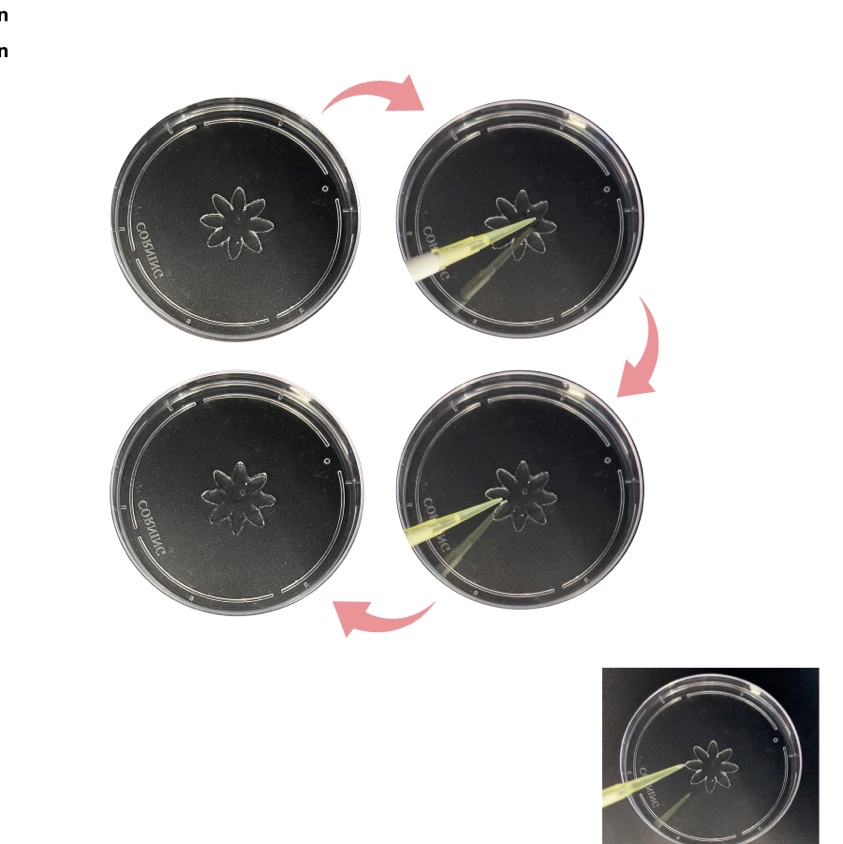


**Figure S8.** Shape response of the gel after the addition of an equivalent amount of pure water, showing no noticeable mechanical deformation.


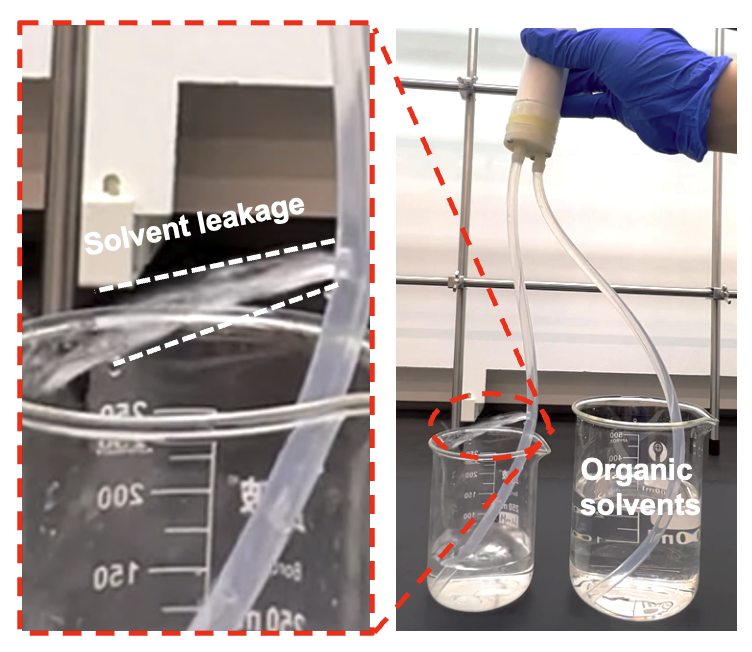


**Figure S9.** Photograph of the experimental pipeline model used to simulate organic solvent leakage under dynamic flow conditions.

**Table S1.** Summary of the decay information upon the biexponential fitting results of the emission decay spectra in the case of aggregated-Au_18_ NCs and Au_18_-based gel.


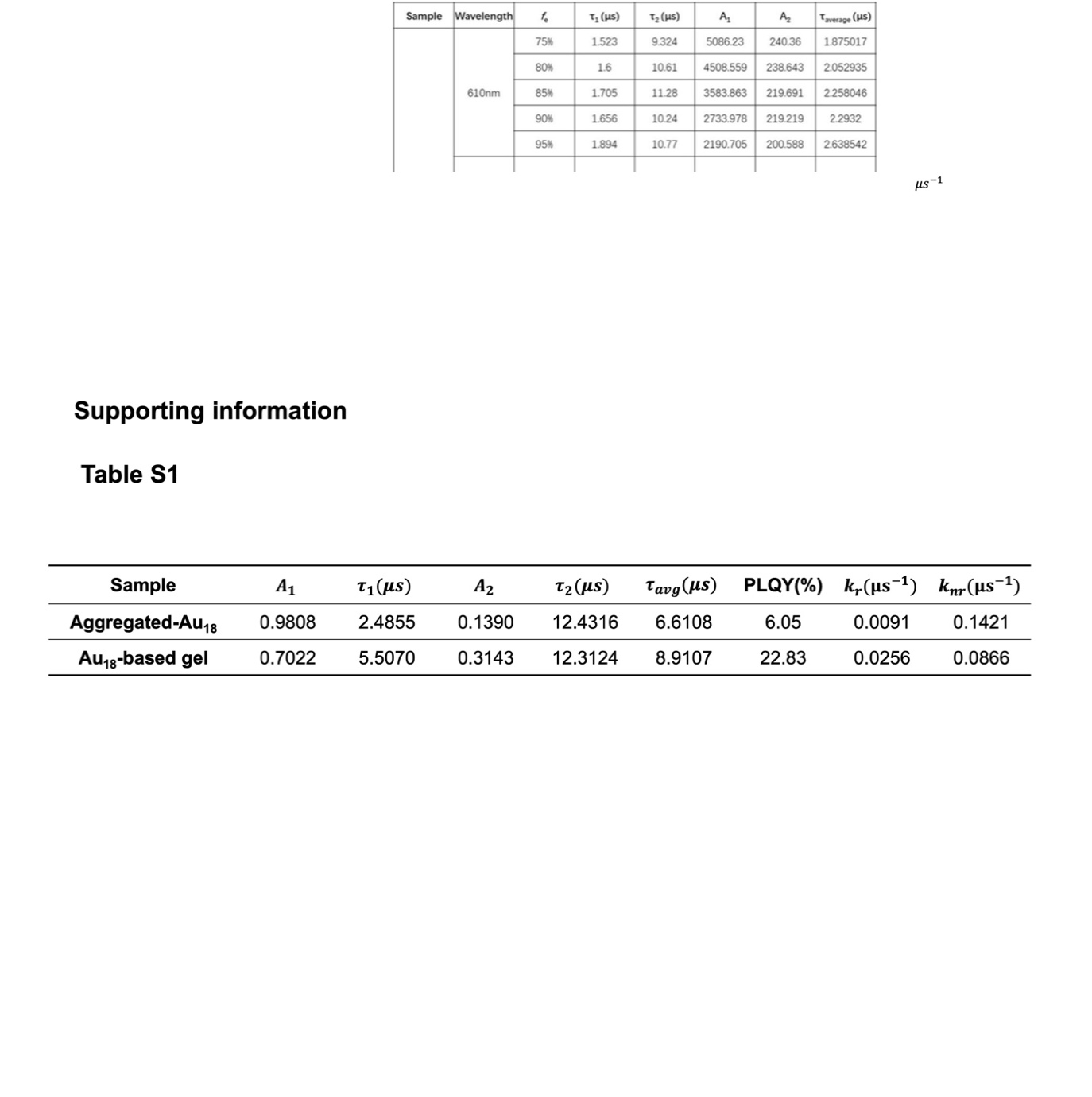


To investigate the underlying luminescent mechanism, the measured photoluminescent lifetime of aggregated Au_18_ in liquid phase and deswollen Au_18_-based gel were performed a second-order exponential decay fit. The fitting results including the average PL lifetime ($\tau_{avg})$, the radiative decay (*k_r_*) and the non-radiative decay rate (*k_nr_*) were summarized in Table S1. These results show that in the deswollen Au_18_ gel phase, the *k_r_* is significantly enhanced to 0.0256 µs⁻¹, while the *k_nr_* is reduced to 0.0866 µs⁻¹, resulting in a PLQY of 22.83% and a PL lifetime of 8.91 µs. In contrast, in the liquid phase of aggregated Au_18_, *k_r_* is lower at 0.0091 µs⁻¹, and *k_nr_* is much higher at 0.1421 µs⁻¹, leading to a lower PLQY of 6.05% and a shorter PL lifetime of 6.61 µs. These remarkable differences reveal that the suppressed non-radiative decay and enhanced radiative transitions from liquid phase to gel phase.

**Table S2.** Summary of the decay information upon the biexponential fitting results of the gel during the deswelling process.


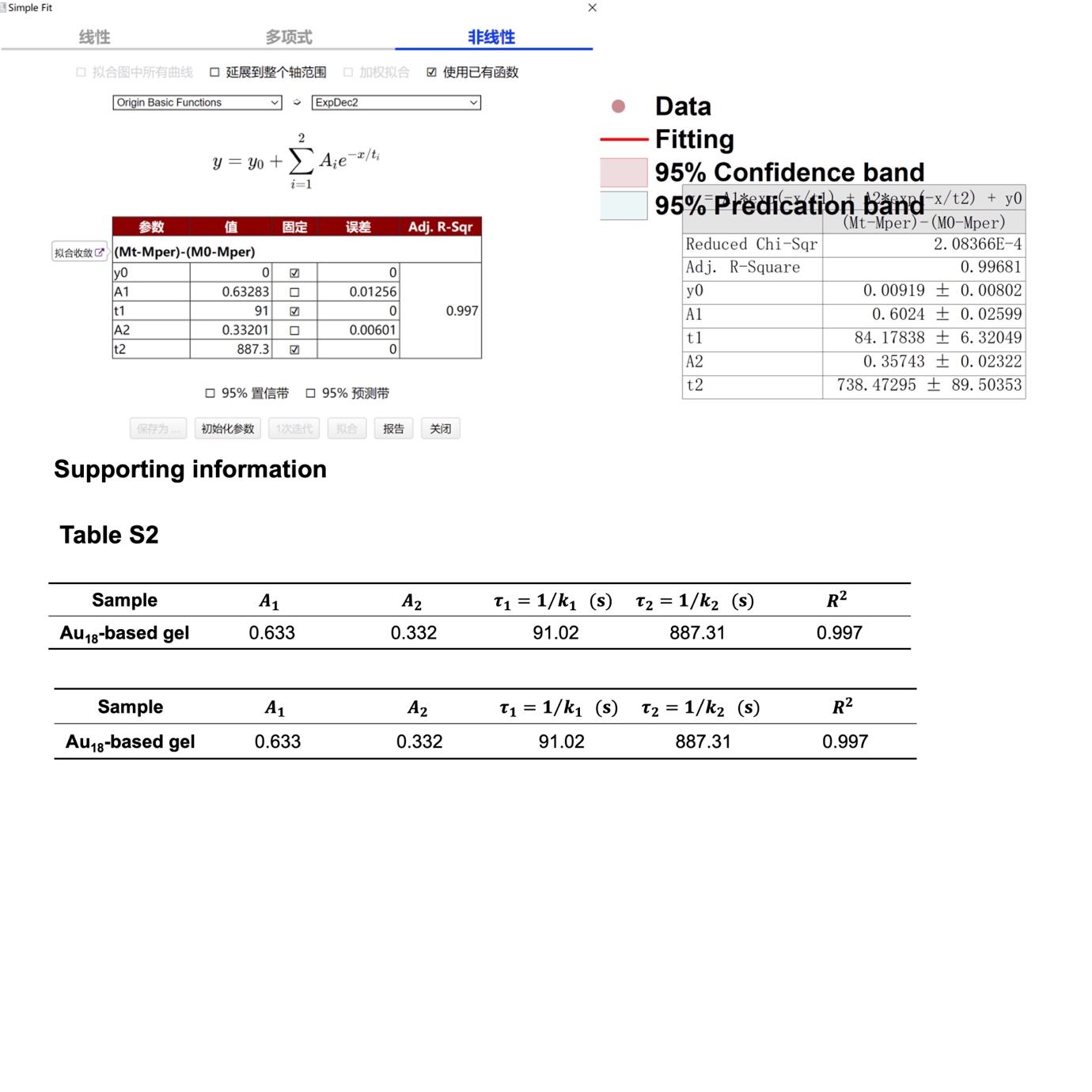

Supplement: Supplementary file 1 — Supporting File 1: adma73386‐sup‐0001‐SuppMat.docx. [file ADMA-38-e73386-s002.docx]
